# Evaluation of portable near-infrared spectroscopy for authentication of mRNA based COVID-19 vaccines

**DOI:** 10.1371/journal.pone.0267214

**Published:** 2022-05-04

**Authors:** Sulaf Assi, Basel Arafat, Ismail Abbas, Kieran Evans

**Affiliations:** 1 Pharmacy and Biomolecular Sciences, Liverpool John Moores University, Liverpool, United Kingdom; 2 Faculty of Health, Education, Medicine and Social Care, Chelmsford, United Kingdom; 3 Faculty of Science, Lebanese University, Beirut, Lebanon; 4 Perkin Elmer, Buckinghamshire, United Kingdom; Accra Technical University, GHANA

## Abstract

Since its identification in 2019, Covid-19 has spread to become a global pandemic. Until now, vaccination in its different forms proves to be the most effective measure to control the outbreak and lower the burden of the disease on healthcare systems. This arena has become a prime target to criminal networks that spread counterfeit Covid-19 vaccines across the supply chain mainly for profit. Counterfeit vaccines provide false sense of security to individuals, heightens the risk of exposure and outbreak of the virus, and increase the risk of harm linked to Covid-19 infection. Moreover, the increase in counterfeit vaccines feeds hesitancy towards vaccination and erodes the trust in mass immunisation programmes. It is therefore of paramount importance to work on rapid and reliable methods for vaccine authentication. Subsequently this work utilised a portable and non-destructive near infrared (NIR) spectroscopic method for authentication of Covid-19 vaccines. A total of 405 Covid-19 vaccines samples, alongside their main constituents, were measured as received through glass vials. Spectral quality and bands were inspected by considering the raw spectra of the vaccines. Authentication was explored by applying principal component analysis (PCA) to the multiplicative scatter correction-first derivative spectra. The results showed that NIR spectra of the vaccine featured mainly bands corresponding to the mRNA active ingredient. Fewer bands corresponded to the excipients and protein spectra. The vaccines NIR spectra were strongly absorbing with maximum absorbances up to 2.7 absorbance units and that differentiated them from samples containing normal saline only (constituent reported for counterfeit Covid-19 vaccines). Clustering based on PCA offered optimal authentication of Covid-19 vaccines when applied over the range of 9000–4000 cm^-1^These findings shed light on the potential of using NIR for analysing Covid-19 vaccines and presents a rapid and effective initial technique for Covid-19 vaccine authentication.

## Introduction

Covid-19, a new coronavirus linked to cluster pneumonia, was discovered in December 2019 in Wuhan (China) and has quickly spread worldwide establishing a pandemic [1, 2]. Since then, Covid-19 has impacted morbidity and mortality rates worldwide [3]. The reported short- and long-term effects of Covid-19 had affected multiple organs related to cardiovascular, respiratory, gastrointestinal, endocrine and nervous systems [4, 5].

Considering the virulence of Covid-19, vaccination represented the most effective approach for handling it. This latter finding resulted in the rapid development of efficient Covid-19 vaccines [6, 7]. Vaccines are biologics that provide dynamic, adaptable immunity to specific illnesses and contain agents that mimic the microorganisms that cause the infection [8]. Vaccines come in a variety of forms, all of which are designed to teach the immune system how to combat viruses that have infiltrated the human body. Subunit, live-attenuated, recombinant, toxoid, inactivated and conjugated vaccines are among the four types of vaccinations available.

With the emergency approval of mRNA Covid-19 vaccinations, there is now greater push than ever for mRNA to be employed as a modality for various therapeutic techniques, such as treating uncommon genetic illnesses through gene substitution and gene editing [9, 10]. In most vaccine formulations, the viral spike protein is expressed and delivered to the host immune system via lipid nanoparticles, inducing an immunological response that defends against Covid-19 [11]. The use of lipid-based nanoparticles contributes further to improving the bioavailability of the vaccine [12].

Since the increased popularity of vaccines, incidents of counterfeit vaccines have been reported in numerous countries including China, Honduras, Kolkata, Mexico, Mumbai, Mexico, Poland and South Africa [13–18]. In the reported cases, normal saline was used in counterfeit vaccine samples that contained no other active ingredient. In such cases, the impact of receiving counterfeit vaccines extends beyond the treatment ineffectiveness into long-term multiple morbidities or even lethal effects linked to severe disease.

This urges the need for development of methods to authenticate Covid-19 vaccines and detect counterfeit ones. It is noteworthy to mention that authenticating the vaccines encompasses not only characterising its chemical constituents but also its physical properties. Most traditional analytical techniques used for authentication (e.g. high performance liquid chromatography) are time-consuming, costly and require a significant amount of manual labour. Moreover, none of the traditional analytical techniques characterises physical and chemical properties synchronously.

In this sense, near-infrared (NIR) spectroscopy addresses the latter limitation of traditional analytical techniques by being rapid, non-destructive, portable and able to characterise the physicochemical properties of samples within minutes of measurement. NIR spectroscopy also permits measurements to be taken straight through transparent sample containers, such as glass and plastic. This allows for the monitoring of a huge number of samples in the manufacturing line without impacting the line’s throughput. Subsequently, NIRS has established itself as one of the most reliable spectroscopic methods for determining chemical and physical properties of foods [19], pharmaceuticals [20], yellow fever vaccine [21], fuel industry [22], polymers [23] and *Escherichia coli* [24].

NIR spectroscopy is based on the concept that the molecular chemical bonds inside a sample cause significant light absorption at specific vibrational frequencies [25]. The overtones of CH, OH, NH, and SH stretching vibrations, as well as stretching–bending combinations involving these functional groups, are responsible for the NIRS absorption. Between the infrared and visible spectrums, the NIR spectrum ranges from 2500 nm to 800 nm (equivalent to 12000–4000 cm^-1^), and the spectral signal can be employed for advanced analytics [26]. The physical and chemical molecular information cannot usually be derived directly from NIR spectra because it consists of several bands resulting from overtones and combination modes that overlap substantially. For this purpose, NIR spectra combined with chemometric methods (such as principal component analysis (PCA)) can produce relatively quick and precise findings [27–30].

As a result, the purpose of this research was to develop a NIRS analytical approach that could be used in conjunction with PCA for authentication of COVID-19 vaccinations. This approach, we believe, would provide rapid, non-destructive alternative to existing quality control procedures for COVID-19 vaccines.

## Materials and methods

### Materials

405 COVID-19 vaccines based on COVID mRNA were used in this study and were of five batches as follows: batch 1 (n = 60); batch 2 (n = 53); batch 3 (n = 174); batch 4 (n = 54); batch 5 (n = 23) and batch 6 (n = 41). Vaccine samples were obtained through the NHS Central Liverpool Primary Care Network. Vaccines were transported in sealed containers that had ice packs and that maintained the temperature at 2–8⁰C. The collection site was 7 minutes away by car from the laboratory and that minimised the implications of transport on the samples. It is noteworthy to mention that vaccine samples used included left over from reconstituted vials (in normal saline) after six doses had been given to patients. The aforementioned vials included small amount (less than 0.45 mL) that had not been sufficient for medical use. In addition, common excipients present in COVID-19 vaccines and normal saline were purchased from chemical suppliers.

### Instrumentation

**Fig 1:**
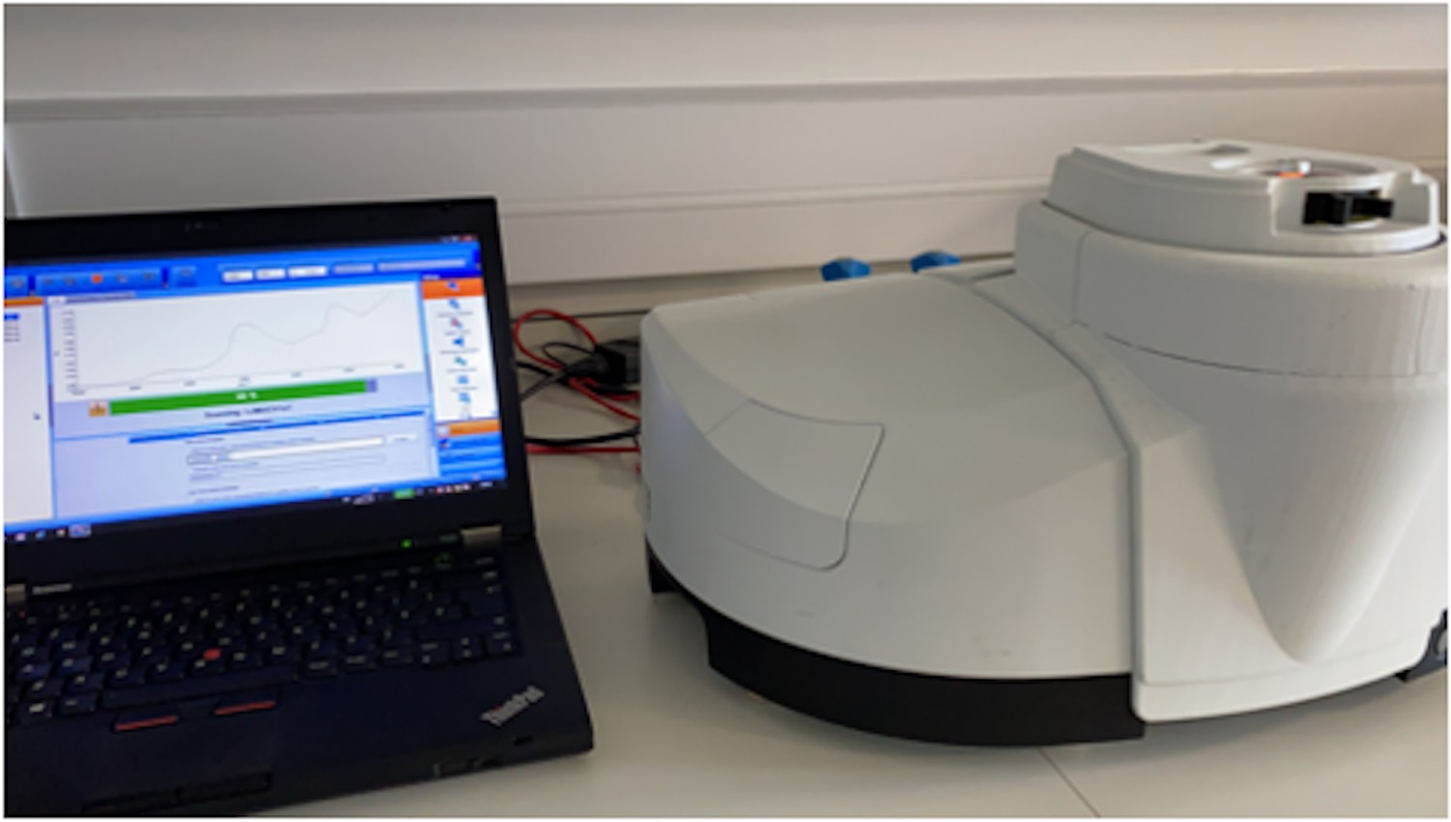
Perkin Elmer Spectrum Two NIR spectrometer.

NIR measurements were conducted using the Perkin Elmer Spectrum Two NIR spectrometer in diffuse reflectance mode (Fig 1).

### Procedure

Each sample had been labelled with Liverpool John Moores University (LJMU) code and stored in freezer prior to measurement. All the required health and safety precautions were followed during labelling and storage procedures according to LJMU risk assessment procedures. Samples were measured as received through glass vials. Three spectra were taken per vial such that each spectrum was the sum of 32 scans over the wavenumber range of 10,000–4000 cm^-1^ and resolution of 8 cm^-1^.

### Data analysis

Spectra were exported into Matlab 2019a where data analysis was applied. Data analysis encompassed assessing spectral quality and identification potential of NIR for vaccines. Spectral quality was evaluated considering four parameters namely the number of bands (n), maximum band intensity and signal to noise ratio (SNR) [31]. Identification potential was evaluated using PCA that was applied to the multiplicative scatter corrected and first derivative (MSC-D1) treated spectra.

## Results and discussion

### NIR activity of vaccines and constituents

When interpreting NIR spectra, numerous factors should be considered that are not limited to the chemical structures of the measured samples. Hence, NIR spectra are sensitive to sample physical properties (e.g. particle size), sample presentation, sample temperature, light penetration and moisture content [32]. The aforementioned factors were taken into account when obtaining NIR spectra of frozen samples and their constituents. Moreover, using diffuse reflectance mode allowed the light to pass through the sample where a more representative spectrum was obtained.

**Fig 2:**
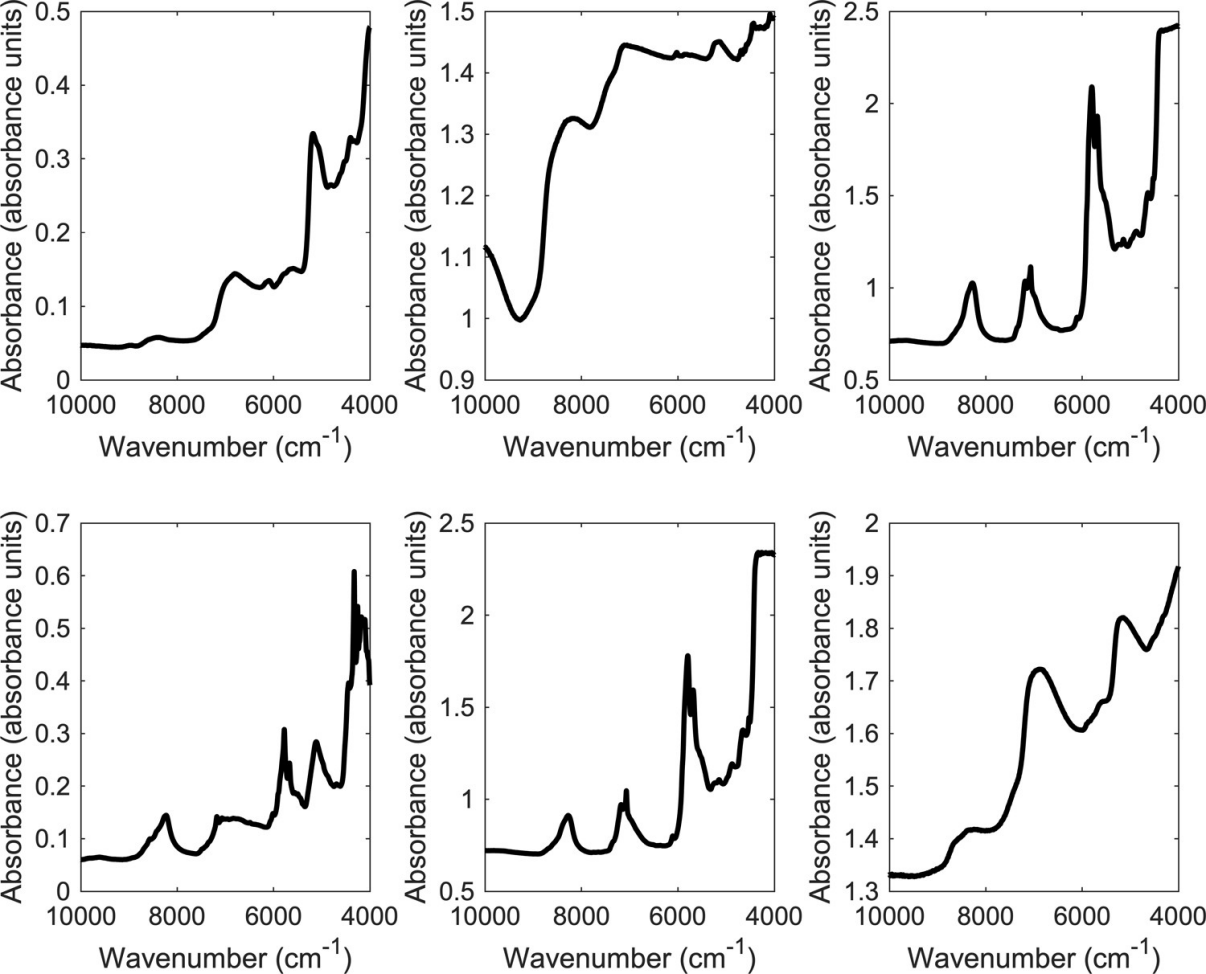
Raw NIR spectrum for (a) mRNA, (b) normal saline solution, (c) excipient 1, (d) excipient 2, (e) excipient 3 and (f) COVID-19 vaccine sample measured using the Perkin Elmer Spectrum II NIR spectrometer in diffuse reflectance mode.

Prior to evaluating the samples, RNA sodium spectra were collected using the Perkin Elmer Spectrum Two NIR spectrometer and showed absorbance values between 0.05 and 0.5 absorbance units over the wavenumber range of 10000–4000 cm^-1^ (Fig 2). The contribution in the aforementioned range was mainly due to the first overtone and binary combinations [33]. The NIR spectrum of RNA sodium showed characteristic bands at 8956, 8432, 6784, 6108, 5648, 5178, 4808 and 4394 cm^-1^. Based on previous studies, the region between 7500–5500 cm^-1^ results mostly from the stretching mode NH and NH_2_ (first overtone and combination bands) present in the nucleic acids of the RNA. Below 5500 cm^-1^, combination bands from stretching and bending of NH_2,_ NH and CH were also observed. The band at 5178 cm^-1^ could be attributed to the C = O stretching vibration (second overtone) that is more prominent in Raman than NIRS [34].

Excipients based on PEGlayted lipids showed bands between 9000–4000 cm^-1^ in the absorbance range of 0.05–2.7 absorbance units. Characteristic bands corresponding to excipients were shown around 8220; 7206, 7078, 5818, 5672, 4900, 4662 and 4120 cm^-1^. Bands between 7700–5500 corresponded to the first overtones of XH stretching modes; whereas bands around 5000–4000 cm^-1^ correspond to combination of CH, OH and NH stretching modes [35].

When vaccine samples were measured, characteristic bands were observed in the range of 9000–4000 cm^-1^. RNA bands were featured in the vaccines’ spectra where bands were seen around 8640, 8354, 6920, 5860, 5170, 4400 and 4330 cm^-1^ (S1 Table). Hence, RNA bands were featured in the vaccines’ spectra in contrary to excipients’ bands that were not featured in the spectra. The slight shift in the bands’ positions between individual samples were related to the differences in water content between vaccines that impacted their physicochemical properties. Hence, vaccines that were more diluted than others showed more prominent bands related to water. Water bands were seen between 5263 and 7143 cm^-1^ that corresponded to free and bound OH groups [36–38].

### Spectral quality of vaccines

**Fig 3:**
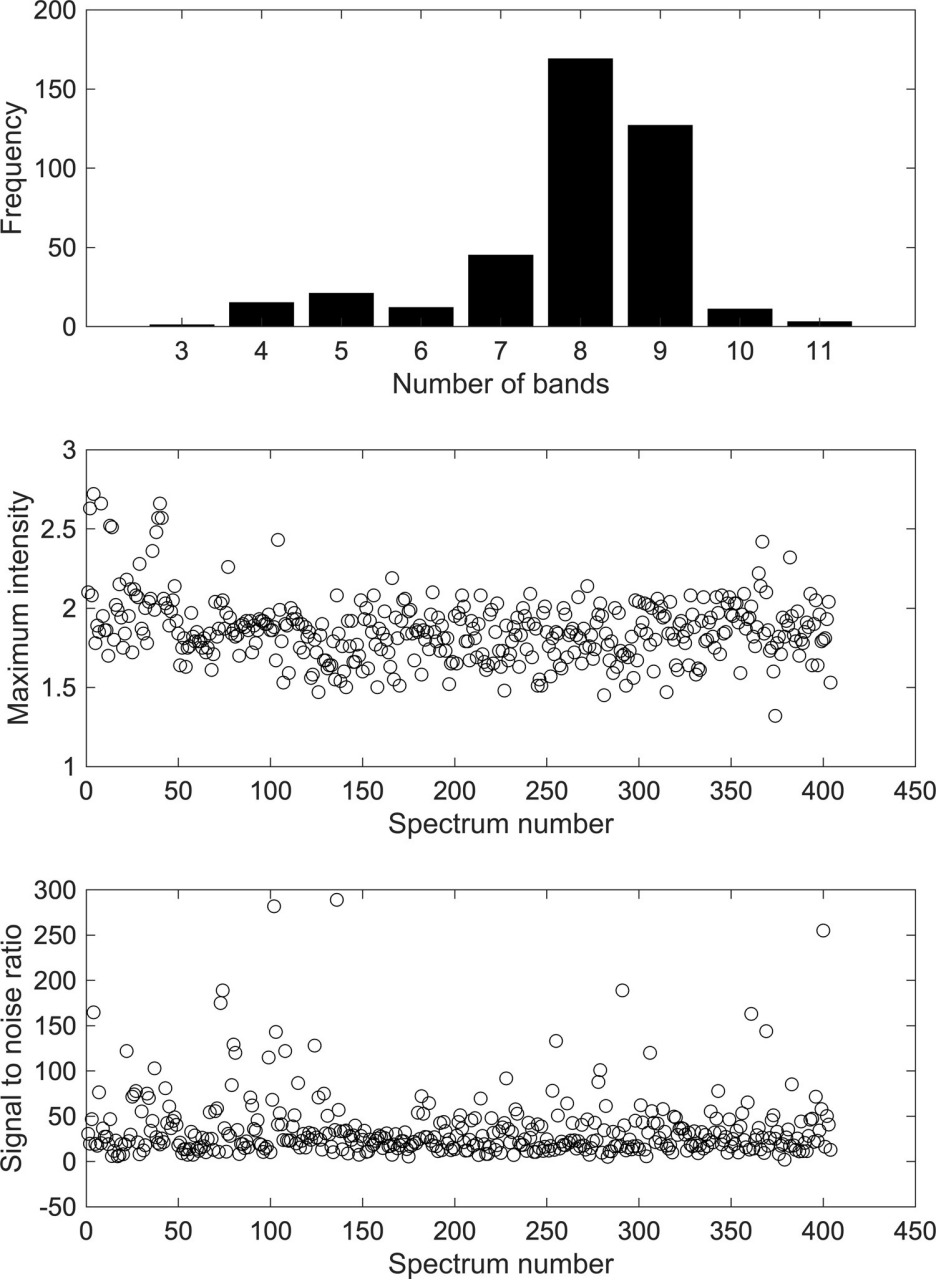
Histogram regarding the number of bands for the measured COVID-19 vaccines (top), maximum peak intensity of the different vaccines (middle) and signal to noise ratio of the vaccines (bottom).

Spectral quality showed strong NIR activity for the majority of the measured samples whereas fewer samples showed weak or medium NIR activity (S2 Table). This was apparent in the numerous absorption bands, maximum intensity and high SNR values (Fig 3). The majority of vaccines showed 7–9 absorption bands with 45 vaccines showing seven bands, 169 showed eight bands and 127 showed nine bands. Only 15 samples showed absorption bands of 10 or 11. On the other hand, 49 samples showed six absorption bands or below. The maximum absorbance intensity of the measured samples was in the range of 1.3–2.7 absorbance units. The majority of the vaccine samples showed maxima absorbances in the range of 1.5–2 absorbance units. The median absorbance of the samples was 1.85 (IQR 1.74–1.96). Nonetheless, not in all cases the high absorbance values indicated better quality where the SNR ratios ranged between 2.05 to 289. SNR of 2.05 was seen for LJMUCV431 that showed six absorbance bands and high maximum intensity of 1.82. In this case, the low SNR obtained indicated that the high intensity was encountered with high noise and weak NIR activity. Only 29 samples showed SNR below 10; where the remaining samples showed SNR of 10 or above that indicated medium or strong NIR activity. The median SNR was 24.2 (IQR 16.7–40.5).

### Authentication of vaccine samples

For vaccine authentication, unsupervised clustering was applied to the MSC-D1 spectra of the vaccines and constituents using principal component analysis (PCA) [39]. The accuracy of clustering was evaluated considering type I and type II errors [40]. Type I errors were encountered when the PC scores of the vaccines were not clustered together. Type II errors were seen when the PC scores of mRNA or constituents (e.g. excipients) were clustered with the vaccine scores. It is worth noting that water bands in the NIR spectra of all measured samples were represented at two key bands, 5304 and 7166 cm^-1^, where MSCD1 alters the position of the bands in NIR. Subsequently, four different wavenumber ranges were compared in relation to datapoints inclusion for clustering as follows:

- The first range included the full wavenumber range
- The second range included the wavenumbers between 4100 and 4734 cm^-1^
- The third considered the wavenumber range between 5488 and 6128 cm^-1^
- The fourth comprised the wavenumber range of 8600 and 8964 cm^-1^

**Fig 4:**
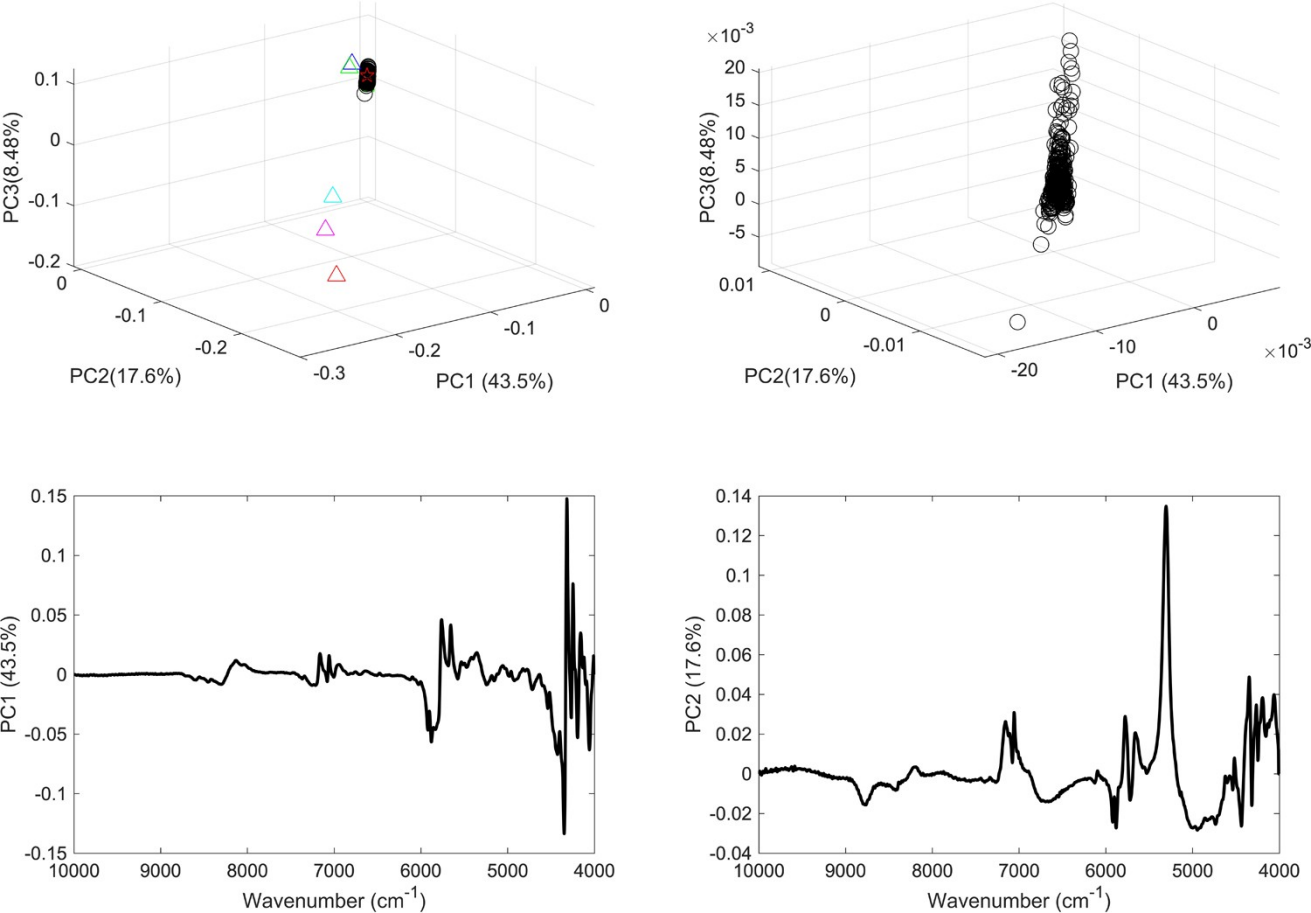
(a) PCA scores plot of COVID-19 vaccine (black circles), mRNA (red star), purified RNA (green and blue stars), normal saline solution (red and green squares) and excipients 1–5 (blue, red, green, cyan and magenta triangles); (b) PCA scores plot of COVID 19 vaccines; (c) PCA loading plot of vaccines and constituents and (d) PCA loading plot of vaccines. The models were applied considering the full wavenumber range.

**Fig 5:**
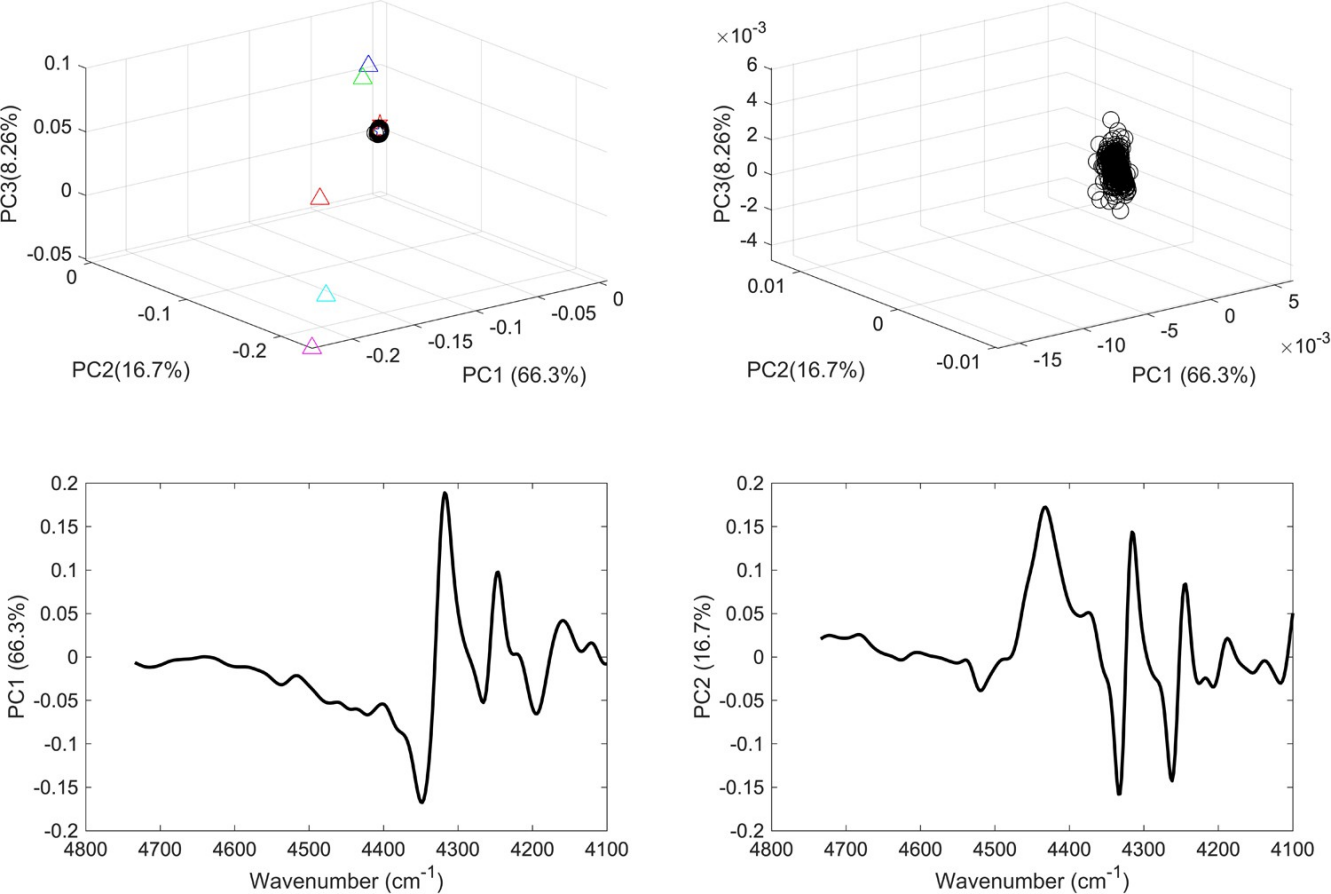
(a) PCA scores plot of COVID-19 vaccine (black circles), mRNA (red star), purified RNA (green and blue stars), normal saline solution (red and green squares) and excipients 1–5 (blue, red, green, cyan and magenta triangles); (b) PCA scores plot of COVID 19 vaccines; (c) PCA loading plot of vaccines and constituents and (d) PCA loading plot of vaccines. The models were applied considering the wavenumber range of 4100–4736 cm^-1^.

**Fig 6:**
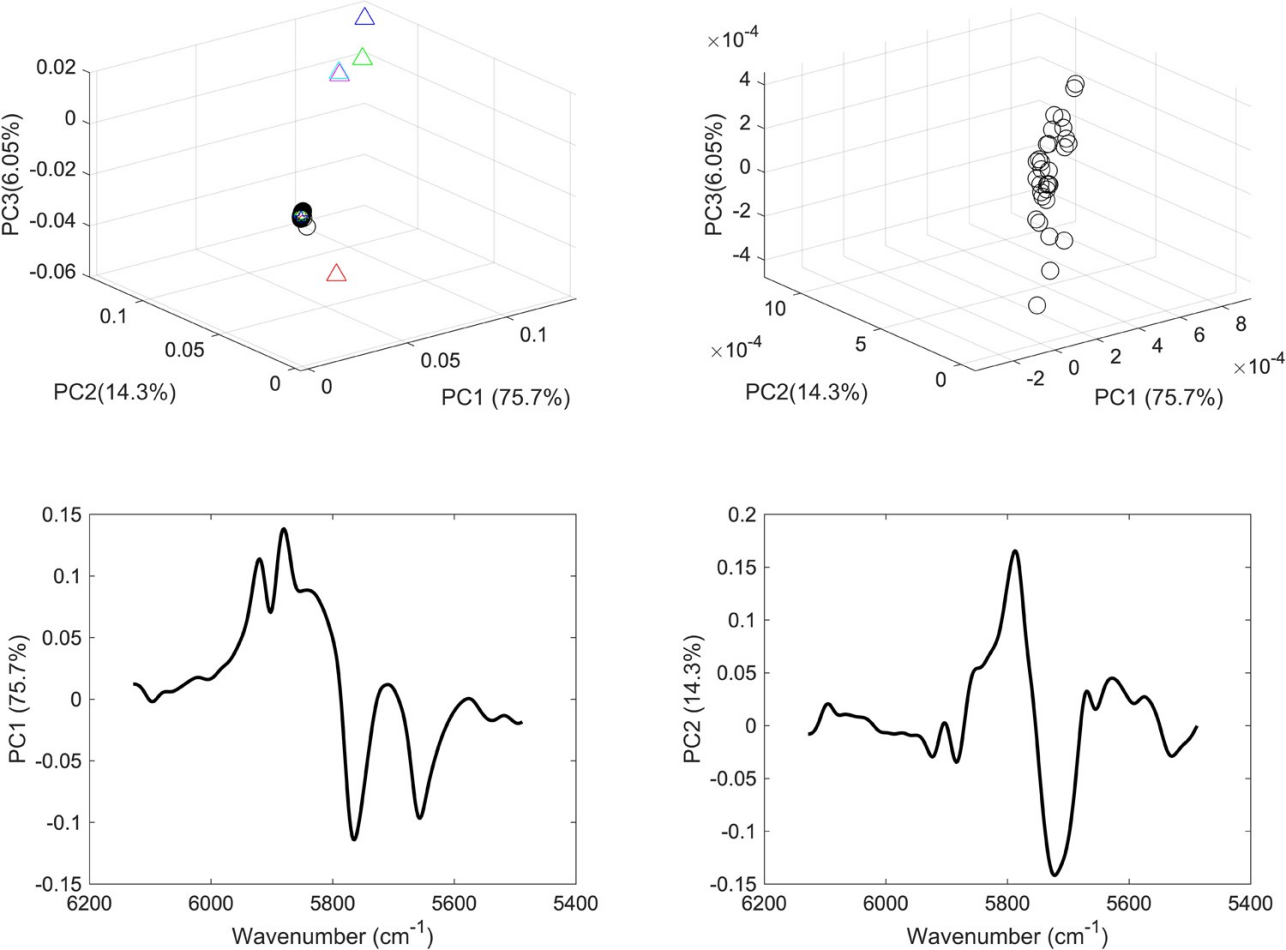
(a) PCA scores plot of COVID-19 vaccine (black circles), mRNA (red star), purified RNA (green and blue stars), normal saline solution (red and green squares) and excipients 1–5 (blue, red, green, cyan and magenta triangles); (b) PCA scores plot of COVID 19 vaccines; (c) PCA loading plot of vaccines and constituents and (d) PCA loading plot of vaccines. The models were applied considering the wavenumber range of 5488–6128 cm^-1^.

**Fig 7:**
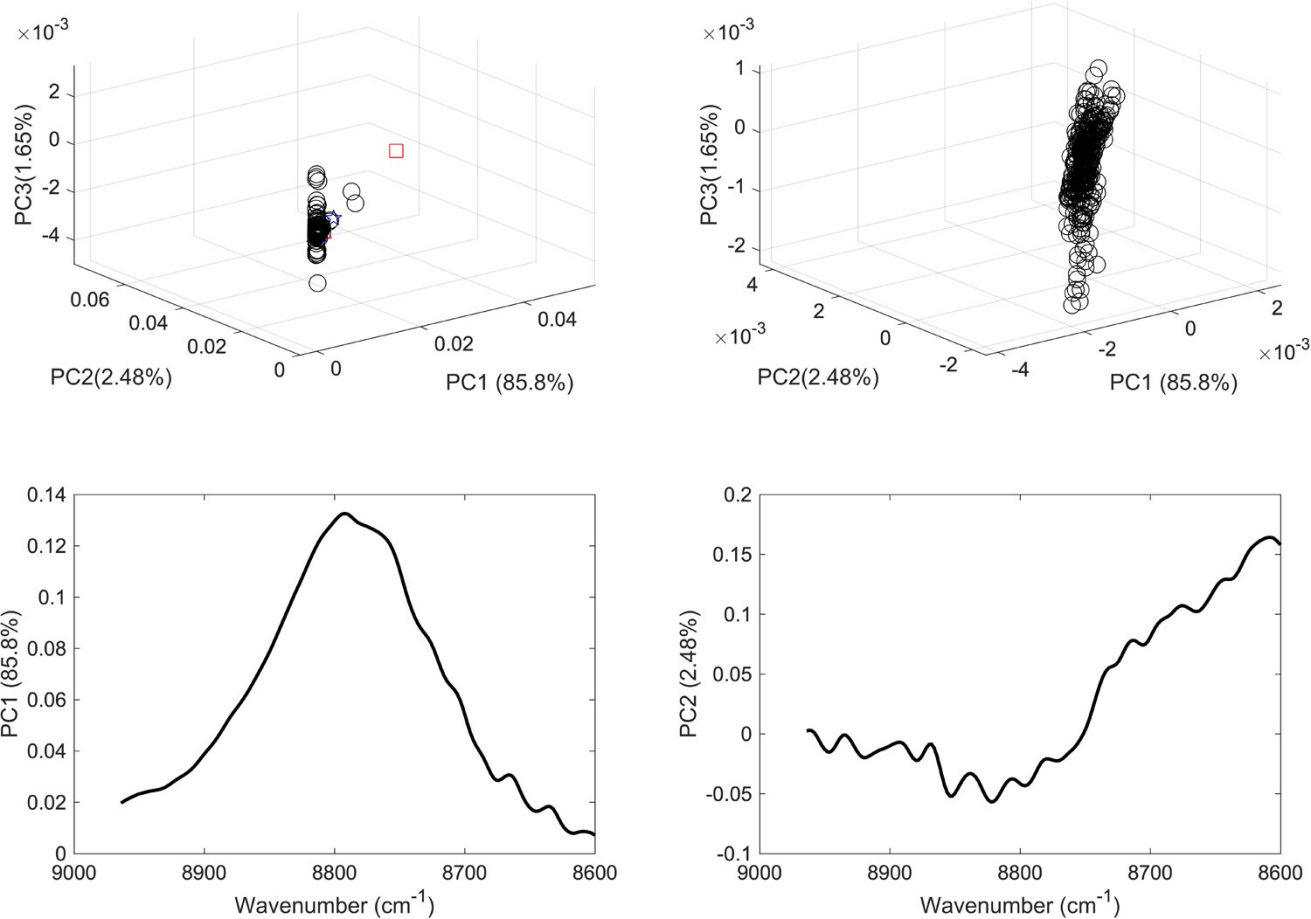
(a) PCA scores plot of COVID-19 vaccine (black circles), mRNA (red star), purified RNA (green and blue stars), normal saline solution (red and green squares) and excipients 1–5 (blue, red, green, cyan and magenta triangles); (b) PCA scores plot of COVID 19 vaccines; (c) PCA loading plot of vaccines and constituents and (d) PCA loading plot of vaccines. The models were applied considering the wavenumber range of 8600–8964 cm^-1^.

For each range, two unsupervised PCA models were applied: the first incorporated the vaccine sample, mRNA, normal saline samples and commonly present excipients in vaccines; and the second included vaccine samples only. Hence models 1 and 2 comprised PCA models applied to the spectral over the full range (Fig 4) models 3 and 4 were PCA models applied to the second range (Fig 5), models 5 and 6 over the third range (Fig 6), and models 7 and 8 over the fourth range (Fig 7).

Model 1 showed distinct clustering of the vaccines’ scores from excipients’ scores but an overlap between the vaccines scores and the mRNA scores. This latter overlap indicated a type II error because the vaccine contained additional constituents than mRNA that were not picked up by the model. The overlap in scores could be attributed to the common spectral features between the vaccine and mRNA (Fig 4).

Both the vaccines and mRNA scores contributed to high variance in the model featured by their spatial position at PC1 that contributed to 43.5% of the variance. The PC1 loading in this case was examined and showed significant spectral bands that were seen in mRNA spectrum. It is noteworthy to mention that the bands related to water content were seen in PC2 loading and not PC1. Moreover, PC2 loading showed other spectral bands which contributed to only 17.6% of the variance among the data. Model 2 PC scores showed the same variance among the data as model 1 that all summed up to 69.6% of the variance among the data and that in turns confirmed the minimum contribution from the constituents’ spectra. Model 2 showed type I error with one score (out of 405) clustered separately from the other PC scores. The loadings on model 2 showed similar features to that of model 1 and that further confirmed the main contribution of mRNA to the spectra of the vaccines. However, this did not exclude the effect of water content on the model that was proven when models 3 and 4 were constructed without including the water content bands (Fig 5).

In this respect, models 3 and 4 PCs contributed to 91% of the variance among the data. Though more variance was captured, an overlap was seen among the scores of the vaccines and mRNA indicating the same type II error as seen in model 1. Nonetheless model 4 did not show any type I error where all the vaccines’ scores were clustered together. This showed that the combination bands (in the range 4100 to 4734 cm^-1^) of NH_2_, NH and OH present in the nucleic acids of the mRNA showed significant contribution in the model and offered more accurate clustering than the full range. Hence the accuracy of clustering depended to a degree on the spectral region considered. For instance, the first overtone region (5488–6128 cm^-1^) that was used in models 5 and 6 showed less accurate clustering of the vaccines despite capturing more variance among the data (Fig 6).

Hence, the variance of the first three PCs in models 5 and 6 added up to 96.1% and showed significant contribution from mRNA and vaccines. However, type I error was higher in model 6 which showed differences in the spatial positions of the vaccines scores. Likewise, low accuracy was seen in models 7 and 8 (over the range 8600–8964 cm^-1^). Hence, both type I and type II errors were encountered in the latter two modes where not all the vaccines’ scores were clustered together and showed overlap with the excipients’ scores (Fig 7).

## Conclusion

In conclusion, NIR spectroscopy demonstrated an effective technique in authenticating COVID-19 vaccines based on mRNA. The spectra of the vaccines featured bands corresponding to mRNA and showed strong absorbance intensities. However, excipients used in the vaccines’ formulation were less featured in their spectra and that affected their authentication. In this respect, clustering based on PCA showed accuracy in differentiating the vaccines from excipients but not from mRNA. The accuracy depended also on the spectral range used prior to clustering where the range of NH_2_, NH and OH combination bands showed more accurate clustering than using the full range.

## Supporting information

S1 Table Main absorption bands of Covid-19 vaccines. (DOCX)

S2 Table Spectral quality of measured Covid-19 vaccines. (DOCX)
