# Supplementary material for: Evaluation of portable near-infrared spectroscopy for authentication of mRNA based COVID-19 vaccines
Source: PLoS One. 2022 May 4;17(5):e0267214. doi: 10.1371/journal.pone.0267214 (PMC9067670; doi:10.1371/journal.pone.0267214)
Supplement: S1 Table — (DOCX) [file pone.0267214.s001.docx]

S1 table. Main peaks of the studied vaccines

| LJMUCVn | Peak Position (cm^-1^) | Peak intensity (absorbance units) |
| --- | --- | --- |
| LJMUCV1 | 8652 8386 6914 5872 5626 5166 4526 4388 4318 | 1.541 1.556 1.987 1.774 1.83 2.126 2.009 2.063 2.096 |
| LMUCV2 | 8636 6894 5602 5172 | 1.032 1.823 1.296 2.626 |
| LMUCV3 | 8646 8376 6932 5872 5620 5172 4530 4396 4336 | 1.649 1.663 1.962 1.854 1.892 2.067 2.03 2.063 2.079 |
| LJMUCV4 | 8574 8330 6886 5600 5146 | 2.129 2.172 2.625 2.671 2.723 |
| LJMUCV5 | 8634 8326 6898 5902 5618 5154 4504 4410 4332 | 1.377 1.399 1.672 1.587 1.625 1.765 1.742 1.761 1.777 |
| LJMUCV6 | 8676 8316 6888 5880 5666 5166 4548 4422 4330 | 1.404 1.442 1.762 1.662 1.689 1.892 1.805 1.825 1.848 |
| LJMUCV7 | 8650 8354 6968 5924 5606 5188 4546 4424 4292 | 1.518 1.532 1.739 1.673 1.709 1.809 1.807 1.829 1.847 |
| LJMUCV8 | 8670 6922 5604 5182 | 1.076 2.21 1.493 2.664 |
| LJMUCV9 | 8660 8338 6944 5896 5618 5196 4526 4396 4332 | 1.553 1.567 1.843 1.738 1.778 1.924 1.901 1.937 1.953 |
| LJMUCV10 | 8648 8268 7064 5878 5746 5174 4406 4324 | 1.587 1.599 1.743 1.734 1.741 1.807 1.85 1.861 |
| LJMUCV11 | 8636 8318 6918 5852 5590 5214 4632 4520 4400 4324 | 1.52 1.533 1.762 1.699 1.727 1.854 1.803 1.83 1.851 1.863 |
| LJMUCV12 | 8662 8326 6952 5568 5192 4526 4312 | 1.222 1.232 1.48 1.372 1.697 1.476 1.524 |
| LJMUCV13 | 8626 8238 6876 5560 5174 | 1.081 1.088 1.868 1.367 2.516 |
| LJMUCV14 | 8598 6854 5584 5150 4178 | 1.193 2.165 1.583 2.511 2.422 |
| LJMUCV15 | 8272 6872 5584 5156 | 1.064 1.576 1.32 1.804 |
| LJMUCV16 | 8196 6878 5846 5644 5182 4520 4398 4272 | 1.581 1.926 1.863 1.887 2.008 1.994 2.011 2.024 |
| LJMUCV17 | 8248 6880 5614 5174 | 0.973 1.646 1.232 1.993 |
| LJMUCV18 | 8556 6880 5604 5152 | 1.091 1.728 1.369 2.15 |
| LJMUCV19 | 8348 6868 5902 5652 5128 4522 4412 4334 | 1.607 1.851 1.785 1.805 1.939 1.907 1.927 1.943 |
| LJMUCV20 | 8342 6904 5874 5626 5196 4530 4382 4330 4238 | 1.517 1.679 1.629 1.637 1.718 1.693 1.723 1.742 1.747 |
| LJMUCV21 | 8126 6828 5868 5180 4524 4390 4330 | 1.472 1.739 1.671 1.836 1.78 1.799 1.813 |
| LJMUCV22 | 8342 6912 5858 5620 5168 4398 4312 | 1.593 1.989 1.804 1.849 2.178 2.067 2.104 |
| LJMUCV23 | 8630 8360 6894 5884 5758 5612 5194 4528 4406 4324 | 1.536 1.549 1.826 1.734 1.748 1.773 1.939 1.927 1.897 1.947 |
| LJMUCV24 | 8632 8254 6888 5640 5202 4326 | 1.521 1.536 1.889 1.773 2.121 2.011 |
| LJMUCV25 | 8260 7106 5192 | 1.495 1.686 1.723 |
| LJMUCV26 | 8364 6900 5880 5640 5196 | 1.673 2.014 1.902 1.943 2.122 |
| LJMUCV27 | 8652 8394 6910 5884 5640 5190 4510 4372 4332 | 1.574 1.594 1.965 1.809 1.859 2.08 1.999 2.038 2.059 |
| LJMUCV28 | 8338 6940 5900 5612 5210 4406 4320 | 1.549 1.918 1.766 1.818 2.065 1.997 2.025 |
| LJMUCV29 | 8688 6926 5580 5184 | 1.039 1.66 1.27 2.275 |
| LJMUCV30 | 8408 6924 5906 5790 5604 5174 4516 4404 4306 | 1.54 1.773 1.695 1.705 1.725 1.868 1.824 1.847 1.863 |
| LJMUCV31 | 8376 6838 5864 5782 5626 5152 4524 4398 4334 4182 | 1.425 1.709 1.613 1.619 1.637 1.825 1.751 1.786 1.806 1.844 |
| LJMUCV32 | 8100 6764 6076 5870 5660 5132 4568 4384 4310 | 1.63 1.877 1.857 1.861 1.856 1.968 1.944 1.977 1.996 |
| LJMUCV33 | 8656 8348 6912 5910 5634 5170 4510 4408 4320 | 1.379 1.391 1.606 1.511 1.538 1.776 1.635 1.657 1.678 |
| LJMUCV34 | 8352 6938 5878 5650 5190 4404 4332 | 1.612 1.902 1.804 1.837 2.024 2.014 2.035 |
| LJMUCV35 | 8412 6888 5908 5602 5182 4544 4402 4292 | 1.633 1.933 1.819 1.865 2.054 1.997 2.041 2.064 |
| LJMUCV36 | 8658 8322 6910 5614 5178 | 0.955 0.962 1.773 1.208 2.359 |
| LJMUCV37 | 8372 6908 5902 5618 5200 4528 4410 4338 | 1.587 1.889 1.774 1.816 1.975 1.938 1.968 1.985 |
| LJMUCV38 | 8626 8396 6840 5606 | 1.271 1.301 2.484 2.417 |
| LJMUCV39 | 8676 6924 5592 5194 | 0.949 1.69 1.199 2.569 |
| LJMUCV40 | 8610 8360 6922 5572 5092 | 1.099 1.108 2.482 1.708 2.663 |
| LJMUCV41 | 8652 6898 5604 5166 | 1.042 1.939 1.351 2.566 |
| LJMUCV42 | 8656 8416 6926 5918 5622 5194 4396 4312 | 1.564 1.583 1.908 1.792 1.843 2.048 2.033 2.056 |
| LJMUCV43 | 8624 8406 6916 5896 5626 5156 4534 4398 4302 | 1.632 1.645 1.902 1.81 1.845 2.019 1.973 2.009 2.029 |
| LJMUCV44 | 8606 8214 6914 5878 5624 5168 4526 4362 4326 | 1.583 1.596 1.85 1.768 1.799 1.978 1.936 1.982 1.999 |
| LJMUCV45 | 8646 8378 6938 5904 5632 5178 4532 4396 4324 | 1.507 1.521 1.778 1.686 1.722 1.889 1.841 1.871 1.888 |
| LJMUCV46 | 8670 8290 6916 5630 5182 4538 4406 4328 | 1.537 1.554 1.831 1.729 1.947 1.911 1.949 1.976 |
| LJMUCV47 | 8680 8378 6906 5896 5608 5168 4494 4362 4322 | 1.591 1.609 1.935 1.805 1.849 2.047 1.996 2.032 2.048 |
| LJMUCV48 | 8626 8364 6902 5872 5628 5182 4542 4376 | 1.591 1.606 1.967 1.83 1.885 2.137 2.061 2.107 |
| LJMUCV49 | 8596 8312 6956 5884 5604 5212 4524 4382 4314 | 1.56 1.57 1.81 1.727 1.763 1.901 1.875 1.906 1.921 |
| LJMUCV50 | 8656 8352 6904 5856 5596 5196 4486 4414 4320 | 1.513 1.525 1.709 1.656 1.679 1.837 1.789 1.809 1.831 |
| LJMUCV51 | 8164 6848 5908 5618 5176 4520 4380 4324 | 1.389 1.547 1.532 1.54 1.616 1.609 1.626 1.635 |
| LJMUCV52 | 9770 8050 7014 5222 4522 | 1.019 1.402 1.664 1.724 1.75 |
| LJMUCV53 | 9748 8136 6898 5870 5668 5196 4516 4404 4280 | 1.414 1.513 1.692 1.677 1.684 1.791 1.767 1.791 1.809 |
| LJMUCV54 | 8214 6870 5914 5636 5176 4520 4400 4306 | 1.367 1.526 1.501 1.513 1.617 1.599 1.615 1.63 |
| LJMUCV55 | 8636 8372 6902 5912 5622 5188 | 1.099 1.115 1.408 1.25 1.284 1.746 |
| LJMUCV56 | 8676 8246 6940 5908 5656 5164 4526 4400 4308 | 1.502 1.519 1.654 1.635 1.647 1.738 1.733 1.751 1.761 |
| LJMUCV57 | 8688 8364 6918 5900 5592 5180 4522 4394 4312 | 1.569 1.589 1.861 1.758 1.797 1.966 1.913 1.948 1.967 |
| LJMUCV58 | 8680 8262 6922 5900 5628 5160 4534 4398 4334 | 1.423 1.448 1.661 1.584 1.607 1.82 1.712 1.739 1.758 |
| LJMUCV59 | 8694 8348 6856 5908 5612 5158 4534 4400 4332 | 1.433 1.455 1.67 1.606 1.622 1.779 1.734 1.77 1.789 |
| LJMUCV60 | 8108 6826 5874 5164 4542 4382 4326 | 1.53 1.717 1.694 1.825 1.793 1.827 1.845 |
| LJMUCV61 | 8344 6902 5920 5646 5186 4398 4326 | 1.495 1.704 1.651 1.674 1.768 1.775 1.785 |
| LJMUCV62 |  |  |
| LJMUCV63 | 9798 8028 7024 5220 4544 | 1.02 1.404 1.663 1.725 1.746 |
| LJMUCV64 | 8312 6868 5884 5638 5184 4526 4396 4302 | 1.499 1.667 1.628 1.643 1.773 1.753 1.788 1.807 |
| LJMUCV65 | 8330 6858 5904 5620 5170 4516 4396 4310 | 1.431 1.613 1.559 1.575 1.716 1.67 1.691 1.708 |
| LJMUCV66 | 9748 8046 6846 5888 5624 5146 | 1.281 1.392 1.635 1.598 1.604 1.747 |
| LJMUCV67 | 8126 6828 5862 5632 5158 4488 4390 4312 | 1.472 1.739 1.671 1.682 1.837 1.781 1.799 1.817 |
| LJMUCV68 | 8708 8376 6894 5878 5626 5170 4390 4320 | 1.287 1.305 1.484 1.435 1.454 1.599 1.591 1.605 |
| LJMUCV69 | 8166 6810 5884 5580 5154 4526 4404 4324 | 1.428 1.589 1.556 1.566 1.708 1.661 1.679 1.697 |
| LJMUCV70 | 8660 8404 6854 5870 5622 5186 4554 4400 4318 | 1.562 1.585 1.885 1.803 1.826 1.965 2.027 2.015 2.042 |
| LJMUCV71 | 8680 8362 6920 5866 5634 5192 4524 4398 4314 | 1.437 1.449 1.662 1.569 1.593 1.82 1.708 1.733 1.753 |
| LJMUCV72 | 8664 8402 6896 5882 5636 5182 4510 4388 4326 | 1.245 1.257 1.511 1.413 1.443 1.873 1.639 1.686 1.719 |
| LJMUCV73 | 8650 8388 6948 5876 5654 5176 4532 4392 4334 | 1.559 1.576 1.858 1.767 1.795 2.028 1.935 1.988 2.011 |
| LJMUCV74 | 8674 8364 6952 5908 5636 5182 4540 4414 4338 | 1.612 1.628 1.911 1.82 1.856 2.027 1.991 2.029 2.051 |
| LJMUCV75 | 8644 8380 6936 5904 5654 5196 4538 4400 4336 4252 | 1.423 1.439 1.759 1.621 1.661 1.869 1.777 1.822 1.845 1.861 |
| LJMUCV76 | 8680 8334 6896 5874 5600 5168 | 1.16 1.178 1.577 1.353 1.406 1.973 |
| LJMUCV77 | 8638 6916 5612 5148 | 0.952 1.849 1.269 2.264 |
| LJMUCV78 | 8650 8390 6912 5906 5630 5162 4530 4396 4328 | 1.503 1.523 1.814 1.725 1.763 1.909 1.888 1.919 1.939 |
| LJMUCV79 | 8664 8360 6930 5856 5624 5196 4546 4308 | 1.434 1.446 1.669 1.572 1.596 1.835 1.696 1.729 |
| LJMUCV80 | 8666 8356 6920 5912 5646 5168 4542 4394 4322 | 1.482 1.5 1.748 1.675 1.708 1.862 1.819 1.847 1.859 |
| LJMUCV81 | 8668 8378 6916 5864 5610 5166 4522 | 1.201 1.209 1.486 1.312 1.344 1.821 1.464 |
| LJMUCV82 | 8670 8418 6910 5910 5636 5152 4542 4410 4312 | 1.42 1.434 1.657 1.576 1.605 1.826 1.724 1.75 1.774 |
| LJMUCV83 | 8212 6966 5894 5618 5168 4542 4382 4328 | 1.406 1.594 1.572 1.59 1.684 1.673 1.692 1.7 |
| LJMUCV84 | 8678 8364 6920 5898 5622 5168 4526 4398 4326 | 1.371 1.386 1.668 1.546 1.588 1.894 1.768 1.811 1.842 |
| LJMUCV85 | 8666 8400 6910 5920 5628 5206 4510 4398 4314 | 1.326 1.34 1.629 1.492 1.536 1.898 1.697 1.729 1.766 |
| LJMUCV86 | 8702 8392 6910 5860 5596 5190 4520 4384 4326 | 1.273 1.288 1.546 1.433 1.466 1.884 1.644 1.693 1.727 |
| LJMUCV87 | 8678 8404 6910 5912 5632 5184 4404 4318 | 1.273 1.295 1.654 1.508 1.559 1.919 1.81 1.853 |
| LJMUCV88 | 8628 8382 6924 5916 5626 5200 4498 4396 4328 | 1.358 1.37 1.598 1.509 1.543 1.864 1.709 1.755 1.785 |
| LJMUCV89 | 8652 8350 6910 5876 5640 5168 4540 4376 4334 | 1.534 1.551 1.776 1.721 1.746 1.88 1.868 1.902 1.915 |
| LJMUCV90 | 8672 8352 6898 5880 5658 5200 4532 4356 4294 | 1.401 1.416 1.609 1.559 1.579 1.723 1.679 1.708 1.719 |
| LJMUCV91 | 8654 8374 6932 5910 5622 5196 4530 4400 4324 | 1.522 1.538 1.757 1.703 1.732 1.865 1.841 1.87 1.886 |
| LJMUCV92 | 8652 8380 6904 5858 5828 5162 4536 4380 4312 | 1.418 1.432 1.637 1.594 1.617 1.758 1.732 1.762 1.776 |
| LJMUCV93 | 8626 8352 6922 5894 5616 5180 4526 4402 4322 | 1.454 1.471 1.734 1.651 1.664 1.831 1.809 1.842 1.859 |
| LJMUCV94 | 8666 8396 6974 5914 5634 5182 4518 4394 4322 | 1.577 1.593 1.805 1.744 1.771 1.897 1.882 1.91 1.925 |
| LJMUCV95 | 8680 8426 6942 5924 5634 5180 4540 4398 4330 | 1.419 1.433 1.672 1.578 1.614 1.922 1.769 1.815 1.844 |
| LJMUCV96 | 8646 8426 6940 5878 5612 5188 4538 4400 4318 | 1.421 1.433 1.672 1.586 1.615 1.922 1.77 1.814 1.847 |
| LJMUCV97 | 8672 8388 6918 5894 5604 5196 4498 4388 4324 | 1.408 1.423 1.723 1.585 1.635 1.899 1.786 1.818 1.843 |
| LJMUCV98 | 8618 8386 6950 5900 5542 5168 4534 4390 4320 | 1.566 1.576 1.767 1.658 1.669 1.9 1.709 1.726 1.742 |
| LJMUCV99 | 8638 8344 6938 5880 5624 5172 4530 4410 4308 | 1.532 1.548 1.821 1.738 1.776 1.917 1.913 1.937 1.956 |
| LJMUCV100 | 8222 7086 5558 5198 4494 | 1.406 1.774 1.793 1.845 1.859 |
| LJMUCV101 | 8630 8288 6910 5912 5682 5150 4522 4378 4308 | 1.576 1.591 1.732 1.714 1.726 1.826 1.821 1.845 1.858 |
| LJMUCV102 | 8628 8372 6992 5876 5810 5648 5192 4512 4394 4312 4166 | 1.625 1.636 1.747 1.739 1.742 1.748 1.815 1.826 1.852 1.862 1.879 |
| LJMUCV103 | 8638 8340 6896 5874 5608 5166 4512 4402 4322 | 1.327 1.338 1.507 1.462 1.459 1.668 1.609 1.632 1.651 |
| LJMUCV104 | 8612 8374 6812 5590 5182 | 1.236 1.269 2.364 2.292 2.427 |
| LJMUCV105 | 8646 8370 6904 5876 5758 5630 5152 4404 4332 4248 | 1.612 1.628 1.879 1.796 1.806 1.820 1.973 1.958 1.976 1.987 |
| LJMUCV106 | 8606 8384 6898 5844 5632 5206 4534 4394 4322 | 1.392 1.405 1.655 1.561 1.588 1.789 1.703 1.736 1.756 |
| LJMUCV107 | 8604 8348 6908 5880 5634 5166 4532 4406 4330 | 1.164 1.171 1.362 1.286 1.310 1.527 1.426 1.448 1.468 |
| LJMUCV108 | 8660 8376 6954 5874 5634 5178 4540 4404 4326 | 1.444 1.463 1.765 1.657 1.693 1.902 1.823 1.853 1.873 |
| LJMUCV109 | 8650 8374 6890 5874 5604 5166 4498 4404 4338 | 1.299 1.31 1.657 1.474 1.522 1.888 1.715 1.754 1.793 |
| LJMUCV110 | 8602 8386 6914 5912 5740 5604 5176 | 1.216 1.224 1.394 1.322 1.334 1.345 1.593 |
| LJMUCV111 | 8640 8296 6902 5886 5746 5624 5186 4534 4400 4334 | 1.461 1.478 1.767 1.649 1.666 1.687 2.003 1.828 1.864 1.889 |
| LJMUCV112 | 8616 8334 6888 5860 5586 5168 4524 4396 4312 | 1.403 1.416 1.729 1.599 1.641 1.929 1.813 1.783 1.843 |
| LJMUCV113 | 8598 8320 6962 5874 5618 5204 4394 4300 4246 | 1.482 1.498 1.883 1.744 1.786 1.966 1.930 1.946 1.961 |
| LJMUCV114 | 8656 8300 6844 5872 5608 5168 4516 4382 4318 | 1.387 1.402 1.664 1.565 1.595 1.927 1.749 1.786 1.813 |
| LJMUCV115 | 8630 8384 6916 5850 5600 5182 4526 4394 4334 | 1.454 1.469 1.719 1.636 1.665 1.9 1.794 1.827 1.845 |
| LJMUCV116 | 8626 8376 6908 5866 5620 5168 4500 4402 4298 | 1.27 1.293 1.622 1.501 1.5383 1.754 1.67 1.693 1.719 |
| LJMUCV117 | 8610 8386 6882 5868 5586 5198 4514 4400 4304 | 1.248 1.263 1.635 1.454 1.505 1.899 1.699 1.745 1.799 |
| LJMUCV118 | 8600 8372 6928 5862 5596 5180 4518 4400 4316 | 1.404 1.413 1.604 1.546 1.571 1.722 1.678 1.699 1.715 |
| LJMUCV119 | 8674 8350 6908 5868 5588 5152 4388 4320 | 1.289 1.307 1.592 1.464 1.501 1.881 1.716 1.751 |
| LJMUCV120 | 8646 8388 6910 5580 5170 4526 4400 | 1.092 1.099 1.399 1.251 1.821 1.41 1.447 |
| LJMUCV121 | 8418 6890 5880 5604 5154 4528 4400 4326 | 1.527 1.762 1.685 1.711 1.845 1.809 1.831 1.846 |
| LJMUCV122 | 8246 6796 5856 5618 5138 4530 4402 4298 | 1.342 1.48 1.466 1.476 1.536 1.536 1.547 1.556 |
| LJMUCV123 | 8314 6802 5872 5588 5140 4528 4398 4310 | 1.318 1.494 1.455 1.472 1.555 1.553 1.571 1.583 |
| LJMUCV124 | 8344 6922 5870 5652 5174 4536 4400 4328 | 1.601 1.732 1.697 1.703 1.792 1.763 1.785 1.799 |
| LJMUCV125 | 8360 6832 5852 5626 5154 4502 4394 4290 | 1.409 1.609 1.543 1.561 1.716 1.673 1.703 1.723 |
| LJMUCV126 | 8384 6890 5880 5766 5146 4494 4408 4320 | 1.295 1.373 1.368 1.372 1.453 1.443 1.456 1.467 |
| LJMUCV127 | 8232 6974 5778 5660 5188 4518 4394 4320 4232 | 1.539 1.703 1.703 1.699 1.799 1.77 1.808 1.834 1.834 |
| LJMUCV128 | 8256 6822 5844 5626 5118 4546 4402 4324 | 1.449 1.787 1.705 1.729 1.889 1.851 1.879 1.902 |
| LJMUCV129 | 8644 8328 6802 5876 5604 5154 4516 4400 4316 | 1.3557 1.372 1.576 1.521 1.539 1.668 1.635 1.652 1.666 |
| LJMUCV130 | 8398 6898 5876 5600 5168 4518 4404 4326 | 1.425 1.588 1.538 1.557 1.673 1.637 1.654 1.668 |
| LJMUCV131 | 8248 6850 5876 5610 5172 4410 4308 | 1.302 1.514 1.483 1.498 1.595 1.605 1.622 |
| LJMUCV132 | 8332 6870 5910 5636 5166 4394 4296 | 1.338 1.544 1.483 1.505 1.639 1.626 1.642 |
| LJMUCV133 | 8246 6944 5604 5182 4398 4326 4224 | 1.459 1.584 1.572 1.619 1.629 1.639 1.642 |
| LJMUCV134 | 9778 8046 6838 5804 5156 4508 4406 4302 | 1.335 1.461 1.688 1.681 1.763 1.754 1.775 1.791 |
| LJMUCV135 | 8608 8236 6882 5918 5788 5604 5168 | 0.953 0.968 1.275 1.071 1.087 1.113 1.553 |
| LJMUCV136 | 8628 8372 6924 5846 5606 5174 4396 4334 | 1.631 1.646 1.965 1.844 1.882 2.083 2.051 2.072 |
| LJMUCV137 | 8300 6906 5878 5656 5154 4506 4402 4332 | 1.575 1.711 1.695 1.705 1.812 1.799 1.826 1.841 |
| LJMUCV138 | 8328 6896 5868 5638 5168 4490 4404 4312 | 1.329 1.459 1.44 1.448 1.523 1.514 1.528 1.539 |
| LJMUCV139 | 9722 8010 6812 5596 5214 | 0.803 1.205 1.725 1.704 1.764 |
| LJMUCV140 | 8356 6894 5886 5610 5174 4500 4406 4314 | 1.376 1.506 1.483 1.498 1.58 1.578 1.592 1.603 |
| LJMUCV141 | 8318 6856 5866 5624 5168 4498 4396 4318 | 1.314 1.425 1.409 1.417 1.498 1.477 1.49 1.5 |
| LJMUCV142 | 8336 6834 5866 5624 5142 4538 4402 4312 | 1.512 1.797 1.723 1.741 1.918 1.859 1.892 1.916 |
| LJMUCV143 | 8296 6784 6026 5830 5610 5170 4518 4386 4336 | 1.439 1.649 1.6 1.609 1.623 1.762 1.734 1.761 1.775 |
| LJMUCV144 | 8380 6910 5846 5590 5144 4522 4402 4330 | 1.519 1.802 1.717 1.744 1.907 1.862 1.899 1.924 |
| LJMUCV145 | 8338 6914 5844 5636 5184 4512 4402 4310 | 1.375 1.551 1.517 1.531 1.646 1.621 1.64 1.655 |
| LJMUCV146 | 8336 6898 5872 5748 5156 4496 4402 4310 | 1.476 1.553 1.557 1.559 1.615 1.628 1.646 1.656 |
| LJMUCV147 | 8398 6906 5864 5604 5156 4510 4398 4306 | 1.466 1.692 1.615 1.638 1.773 1.715 1.734 1.749 |
| LJMUCV148 | 9742 7988 6846 5586 5126 4508 | 0.798 1.168 1.622 1.6 1.671 1.696 |
| LJMCV149 | 8358 6906 5876 5634 5160 4528 4386 4324 | 1.699 1.934 1.883 1.901 2.013 1.996 2.029 2.048 |
| LJMUCV150 | 8300 6808 5902 5624 5154 4524 4392 4314 | 1.338 1.508 1.472 1.487 1.602 1.574 1.593 1.608 |
| LJMUCV151 | 8250 6840 5876 5706 5182 4538 4398 4312 | 1.598 1.818 1.784 1.789 1.895 1.881 1.915 1,931 |
| LJMUCV152 | 9826 8230 6810 5854 5622 5160 4396 4328 | 1.233 1.385 1.874 1.764 1.792 1.982 1.974 1.996 |
| LJMUCV153 | 9740 8000 6810 5840 5162 4528 4390 4318 | 1.134 1.278 1.525 1.523 1.592 1.592 1.609 1.62 |
| LJMUCV154 | 9678 8072 6840 5892 5614 5160 4536 4396 4316 | 1.402 1.499 1.763 1.731 1.744 1.885 1.869 1.9 1.92 |
| LJMUCV155 | 9768 8082 6850 5858 5618 5168 4392 4328 | 1.322 1.470 1.734 1.734 1.736 1.833 1.837 1.851 |
| LJMUCV156 | 8266 6848 5870 5606 5146 4392 4296 4196 | 1.471 1.893 1.747 1.779 2.079 1.969 2.005 2.068 |
| LMUCV157 | 8354 6908 5890 5610 5200 4528 4396 4278 | 1.481 1.671 1.629 1.65 1.742 1.739 1.759 1.769 |
| LJMUCV158 | 8398 6896 5870 5634 5180 4536 4380 4294 | 1.259 1.429 1.392 1.408 1.503 1.481 1.496 1.504 |
| LJMUCV159 | 8286 6818 5878 5626 5154 4534 4402 4310 | 1.542 1.762 1.709 1.718 1.871 1.815 1.842 1.861 |
| LJMUCV160 | 8384 6854 5882 5754 5154 4390 4322 | 1.522 1.636 1.613 1.618 1.723 1.725 1.735 |
| LJMUCV161 | 8206 6770 5862 5124 4518 4400 4330 4240 | 1.585 1.742 1.732 1.786 1.788 1.816 1.837 1.84 |
| LJMUCV162 | 8304 6866 5842 5608 5132 4532 4406 4330 4172 | 1.513 1.822 1.741 1.763 1.943 1.893 1.926 1.950 1.984 |
| LJMUCV163 | 8394 7448 6806 5878 5750 5626 5170 4518 4368 4314 4204 | 1.558 1.592 1.679 1.675 1.679 1.682 1.751 1.759 1.787 1.799 1.804 |
| LJMUCV164 | 8298 6796 5896 5658 5148 4526 4370 4306 | 1.485 1.625 1.614 1.620 1.689 1.691 1.713 1.724 |
| LJMUCV165 | 9720 8086 6856 5874 5608 5174 4530 4398 4310 | 1.214 1.328 1.525 1.531 1.533 1.601 1.603 1.619 1.629 |
| LJMUCV166 | 8166 6828 5860 5594 5110 4386 4286 | 1.422 2.064 1.902 1.932 2.199 2.148 2.186 |
| LJMUCV167 | 9662 8052 6812 5848 5578 5156 4542 4400 4298 | 1.245 1.325 1.462 1.475 1.468 1.517 1.526 1.539 1.55 |
| LJMUCV168 | 8344 6822 5856 5600 5174 4532 4402 4312 | 1.505 1.743 1.668 1.688 1.806 1.937 1.836 1.866 |
| LJMUCV169 | 8358 8138 6788 5860 5746 5140 4506 4398 4334 | 1.488 1.499 1.698 1.682 1.68 1.785 1.774 1.796 1.811 |
| LJMUCV170 | 9638 8070 6782 5862 5566 5144 4534 4416 4288 | 1.195 1.268 1.417 1.416 1.419 1.484 1.486 1.497 1.513 |
| LJMUCV171 | 8312 6764 5860 5146 4480 4406 4316 4156 | 1.563 1.743 1.724 1.906 1.839 1.864 1.885 1.922 |
| LJMUCV172 | 8190 6844 5864 5638 5170 4542 4380 4322 | 1.576 1.892 1.869 1.877 2.018 1.998 2.032 2.05 |
| LJMUCV173 | 8304 6878 5856 5638 5168 4494 4404 4328 | 1.532 1.917 1.843 1.872 2.024 2.015 2.041 2.058 |
| LJMUCV174 | 8350 6896 5868 5620 5160 4518 4412 4320 | 1.455 1.746 1.655 1.683 1.831 1.799 1.82 1.84 |
| LJMUCV175 | 8338 6886 5574 5164 | 1.373 1.757 1.649 1.981 |
| LJMUCV176 | 8244 6820 5842 5650 5142 4408 4326 | 1.608 1.872 1.834 1.839 1.982 1.967 1.99 |
| LJMUCV177 | 8312 6864 5842 5616 5164 4416 4304 | 1.48 1.721 1.694 1.713 1.812 1.826 1.84 |
| LJMUCV178 | 8300 6868 5862 5650 5178 4492 4364 4312 | 1.328 1.511 1.5 1.518 1.636 1.644 1.668 1.679 |
| LJMUCV179 | 8228 6814 5910 5596 5182 4542 4388 4292 | 1.474 1.732 1.677 1.689 1.855 1.796 1.818 1.839 |
| LJMUCV180 | 8348 6936 5878 5634 5174 4398 4272 | 1.529 1.716 1.67 1.692 1.814 1.835 1.851 |
| LJMUCV188 | 8232 6910 5628 5204 4496 | 1.278 1.834 1.803 1.875 1.886 |
| LJMUCV189 | 8362 6894 5878 5634 5182 4524 4402 4332 | 1.307 1.449 1.422 1.436 1.575 1.531 1.553 1.566 |
| LJMUCV190 | 8368 6898 5878 5610 5186 4538 4402 4306 | 1.622 1.764 1.752 1.76 1.827 1.829 1.849 1.855 |
| LJMUCV191 | 8320 6870 5898 5638 5162 4528 4418 4316 | 1.532 1.698 1.671 1.684 1.799 1.769 1.785 1.801 |
| LJMUCV192 | 8400 6908 5882 5764 5622 5164 4520 4400 4330 | 1.541 1.72 1.681 1.689 1.7 1.829 1.798 1.833 1.85 |
| LJMUCV193 | 8382 6936 5864 5616 5162 4382 4310 | 1.475 1.6 1.598 1.609 1.691 1.729 1.741 |
| LJMUCV194 | 8212 6810 5906 5610 5158 4528 4402 4312 | 1.635 1.842 1.824 1.833 1.925 1.921 1.947 1.961 |
| LJMUCV195 | 9788 8018 6784 5870 5112 4546 4328 | 1.413 1.615 1.975 1.97 2.059 2.06 2.096 |
| LJMUCV196 | 9730 8048 6856 5698 5210 4530 4414 4320 | 1.175 1.427 1.75 1.749 1.824 1.827 1.839 1.852 |
| LJMUCV197 | 8330 6892 5882 5638 5160 4516 4400 4330 | 1.543 1.695 1.672 1.681 1.763 1.759 1.776 1.785 |
| LJMUCV198 | 8310 6902 5882 5622 5176 4518 4394 4312 | 1.533 1.812 1.75 1.772 1.919 1.889 1.922 1.938 |
| LJMUCV199 | 8634 8318 6940 5898 5746 5618 5180 4488 4370 4318 | 1.417 1.428 1.575 1.547 1.558 1.569 1.676 1.694 1.721 1.734 |
| LJMUCV200 | 8152 6882 5878 5596 5170 4500 4404 4310 | 1.529 1.767 1.723 1.74 1.861 1.845 1.868 1.885 |
| LJMUCV201 | 8188 6936 5846 5632 5168 4412 4300 | 1.331 1.579 1.552 1.565 1.663 1.666 1.679 |
| LJMUCV202 | 8382 6856 5876 5620 5164 4392 4332 | 1.435 1.692 1.63 1.653 1.793 1.799 1.813 |
| LJMUCV203 | 8366 6892 5872 5608 5178 4486 4404 4300 | 1.323 1.639 1.559 1.589 1.715 1.697 1.714 1.729 |
| LJMUCV204 | 8306 6844 5880 5624 5154 | 1.395 1.755 1.639 1.674 1.814 |
| LJMUCV205 | 9692 8182 6882 5838 5594 5190 4505 4396 4304 | 1.233 1.291 1.446 1.414 1.421 1.514 1.492 1.508 1.522 |
| LJMUCV206 | 9718 8044 6854 5886 5602 5164 4524 4406 4330 | 1.091 1.239 1.553 1.526 1.526 1.649 1.612 1.633 1.653 |
| LJMUCV207 | 8202 6772 5902 5638 5170 4498 4396 4288 | 1.418 1.559 1.552 1.553 1.624 1.645 1.629 1.657 |
| LJMUCV208 | 8270 6892 5880 5630 5150 4404 4318 | 1.64 1.808 1.799 1.809 1.924 1.935 1.949 |
| LJMUCV209 | 8234 6812 5886 5600 5178 4528 4386 4320 | 1.438 1.562 1.547 1.552 1.626 1.626 1.642 1.651 |
| LJMUCV210 | 8204 7000 5772 5658 5144 4324 4206 | 1.593 1.755 1.8 1.778 1.853 1.967 1.947 |
| LJMUCV211 | 8376 6850 5878 5652 5160 4522 4408 4318 | 1.594 1.782 1.766 1.774 1.901 1.871 1.906 1.929 |
| LJMUCV212 | 8284 6896 5840 5630 5170 4532 4394 4322 | 1.717 1.97 1.939 1.95 2.037 2.042 2.069 2.083 |
| LJMUCV213 | 8236 6860 5856 5604 5208 | 1.407 1.93 1.843 1.876 2.011 |
| LJMUCV214 | 8316 6868 5864 5632 5206 4534 4402 4336 | 1.474 1.709 1.657 1.672 1.771 1.758 1.774 1.785 |
| LJMUCV215 | 8318 6856 5884 5630 5182 4532 4406 4328 | 1.421 1.654 1.581 1.603 1.787 1.713 1.746 1.769 |
| LJMUCV216 | 8398 6876 5890 5624 5176 4512 4420 4328 | 1.345 1.581 1.511 1.536 1.665 1.638 1.654 1.671 |
| LJMUCV217 | 8386 6936 5860 5634 5170 4542 4400 4294 | 1.467 1.815 1.723 1.753 1.911 1.879 1.905 1.924 |
| LJMUCV218 | 8348 6908 5846 5610 5168 4500 4394 4320 | 1.513 1.74 1.657 1.683 1.866 1.788 1.809 1.826 |
| LJMUCV219 | 8330 6912 5892 5594 5214 4506 4368 4328 | 1.529 1.681 1.641 1.66 1.809 1.761 1.784 1.795 |
| LJMUCV220 | 8374 6894 5570 5188 | 1.245 1.635 1.555 1.694 |
| LJMUCV221 | 8368 6970 5890 5622 5222 4534 4354 4320 | 1.527 1.788 1.706 1.737 1.854 1.844 1.888 1.902 |
| LJMUCV222 | 8386 6900 5910 5646 5174 4390 4526 4306 | 1.619 1.964 1.848 1.887 2.072 2.062 2.017 2.083 |
| LJMUCV223 | 8320 6856 5868 5620 5188 4522 4380 4296 | 1.379 1.603 1.567 1.585 1.686 1.677 1.697 1.707 |
| LJMUCV224 | 8340 7006 5908 5646 5188 4512 4404 4334 | 1.441 1.553 1.557 1.57 1.617 1.64 1.655 1.662 |
| LJMUCV225 | 8374 7062 5864 5636 5146 4526 4404 4274 | 1.393 1.481 1.486 1.495 1.544 1.585 1.604 1.611 |
| LJMUCV226 | 8386 6926 5874 5640 5202 4484 4406 4330 | 1.362 1.543 1.501 1.518 1.614 1.609 1.625 1.638 |
| LJMUCV227 | 8356 6924 5880 5618 5188 4516 4380 4326 | 1.564 1.84 1.776 1.807 1.921 1.917 1.943 1.957 |
| LJMUCV228 | 8382 6936 6026 5872 5650 5166 4392 4326 | 1.572 1.827 1.732 1.761 1.781 1.952 1.968 1.989 |
| LJMUCV229 | 8162 6730 5908 5648 5140 4524 4398 4324 | 1.382 1.539 1.529 1.532 1.607 1.61 1.632 1.647 |
| LJMUCV230 | 8318 6962 6014 5868 5646 5192 4398 4326 | 1.565 1.718 1.684 1.704 1.709 1.802 1.836 1.85 |
| LJMUCV231 | 8368 6976 5906 5734 5624 5176 4518 4406 4336 | 1.685 1.904 1.874 1.885 1.895 1.999 1.995 2.021 2.031 |
| LJMUCV232 | 8404 8200 6840 5908 5750 5640 5164 4522 4396 4326 | 1.369 1.376 1.569 1.546 1.557 1.561 1.679 1.68 1.714 1.731 |
| LJMUCV233 | 8308 6886 5860 5600 5172 4534 4374 4294 | 1.386 1.519 1.498 1.508 1.625 1.582 1.602 1.614 |
| LJMUCV234 | 8716 8342 6904 5880 5640 5140 4520 4396 4302 | 1.413 1.427 1.575 1.549 1.157 1.676 1.691 1.721 1.734 |
| LJMUCV235 | 8366 6868 5878 5620 5156 4522 4408 4310 | 1.234 1.344 1.341 1.353 1.432 1.446 1.465 1.479 |
| LJMUCV236 | 8348 6878 5882 5778 5624 5150 4524 4372 4312 | 1.452 1.764 1.706 1.716 1.732 1.859 1.85 1.879 1.893 |
| LJMUCV237 | 8374 8178 6852 5880 5654 5152 4372 4508 4298 | 1.397 1.4 1.523 1.53 1.54 1.609 1.659 1.638 1.669 |
| LJMUCV238 | 8666 8386 6900 5882 5608 5166 4528 4374 4306 | 1.326 1.339 1.547 1.464 1.486 1.577 1.709 1.6 1.622 |
| LJMUCV239 | 8392 6894 6028 5848 5630 5200 4510 4384 4312 | 1.429 1.682 1.619 1.641 1.659 1.759 1.757 1.779 1.788 |
| LJMUCV240 | 8334 6898 6030 5888 5650 5156 4516 4410 4326 | 1.571 1.749 1.697 1.713 1.729 1.842 1.841 1.866 1.883 |
| LJMUCV241 | 8356 6872 5866 5748 5624 5152 4522 4396 4326 | 1.457 1.699 1.629 1.638 1.653 1.824 1.774 1.812 1.832 |
| LJMUCV242 | 8256 6842 5880 5634 5168 4532 4402 4326 | 1.582 1.792 1.751 1.765 1.944 1.875 1.9 1.919 |
| LJMUCV243 | 8388 6882 6014 5878 5638 5144 4506 4406 4310 | 1.294 1.506 1.464 1.477 1.498 1.588 1.598 1.615 1.628 |
| LJMUCV244 | 8354 6830 5866 5618 5168 4526 4420 4328 | 1.519 1.716 1.694 1.706 1.795 1.799 1.812 1.826 |
| LJMUCV245 | 8276 6858 5892 5604 5158 4530 4392 4314 | 1.694 1.882 1.867 1.878 1.968 1.967 1.992 2.002 |
| LJMUCV246 | 8374 6890 5880 5168 4548 4394 4316 | 1.504 1.852 1.753 1.793 1.909 1.936 1.949 |
| LJMUCV247 | 8320 6892 5892 5664 5166 4506 4402 4336 | 1.525 1.6501 1.632 1.64 1.708 1.714 1.73 1.739 |
| LJMUCV248 | 8306 6866 5902 5760 5610 5156 4540 4402 4310 | 1.544 1.776 1.751 1.761 1.767 1.853 1.884 1.854 1.899 |
| LJMUCV249 | 8404 6890 5880 5624 5266 4412 4328 | 1.529 1.978 1.854 1.898 2.085 2.083 2.105 |
| LJMUCV436 | 8500 8226 6896 5612 5206 4530 4414 4320 | 1.297 1.318 1.621 1.566 1.672 1.658 1.673 1.687 |
| LJMUCV286 | 8300 6808 5892 5642 5156 4540 4388 4326 | 1.544 1.775 1.752 1.767 1.853 1.854 1.887 1.899 |
| LJMUCV250 | 8314 6932 5886 5662 5154 4522 4418 4332 | 1.586 1.758 1.734 1.749 1.847 1.841 1.857 1.87 |
| LJMUCV251 | 8224 6828 5824 5600 5162 4520 4380 4320 | 1.272 1.428 1.398 1.413 1.502 1.49 1.504 1.511 |
| LJMUCV252 | 8608 8360 6950 5876 5654 5182 4516 4402 4330 | 1.299 1.312 1.454 1.435 1.448 1.514 1.523 1.539 1.549 |
| LJMUCV253 | 8164 6868 5886 5624 5166 4532 4310 | 1.236 1.433 1.422 1.43 1.487 1.497 1.511 |
| LJMUCV254 | 8268 6886 5852 5622 5166 4404 4322 | 1.451 1.846 1.746 1.771 1.969 1.95 1.97 |
| LJMUCV255 | 8348 6864 5878 5638 5144 4528 4400 4318 | 1.613 1.885 1.819 1.839 1.987 1.949 1.989 2.014 |
| LJMUCV256 | 8196 6894 5872 5610 5170 4538 4416 4318 | 1.364 1.767 1.678 1.709 1.837 1.819 1.836 1.854 |
| LJMUCV257 | 9716 7964 6828 5816 5146 4516 4406 4298 | 1.261 1.413 1.664 1.658 1.723 1.738 1.748 1.76 |
| LJMUCV258 | 8224 6830 5910 5638 5150 4522 4392 4336 | 1.329 1.472 1.453 1.468 1.534 1.539 1.558 1.566 |
| LJMUCV259 | 9698 8016 6874 6014 5668 5144 4414 4290 | 1.374 1.511 1.703 1.707 1.716 1.76 1.798 1.805 |
| LJMUCV260 | 8302 6906 5880 5652 5172 4516 4396 4324 | 1.513 1.622 1.609 1.617 1.698 1.68 1.699 1.706 |
| LJMUCV261 | 8286 6824 5880 5626 5186 4526 4404 4314 | 1.533 1.725 1.697 1.712 1.816 1.809 1.833 1.846 |
| LJMUCV262 | 9720 8120 6850 5890 5622 5170 4516 4398 4304 | 1.424 1.515 1.726 1.719 1.722 1.802 1.809 1.828 1.838 |
| LJMUCV263 | 8292 6856 5884 5642 5166 4522 4370 4316 | 1.379 1.53 1.513 1.525 1.619 1.607 1.628 1.637 |
| LJMUCV264 | 8142 6812 5870 5622 5178 4516 4392 4334 | 1.393 1.533 1.527 1.529 1.587 1.594 1.609 1.616 |
| LJMUCV265 | 9620 8034 6736 6054 5672 5140 4550 4388 4324 | 1.539 1.633 1.878 1.857 1.856 1.967 1.946 1.977 1.997 |
| LJMUCV266 | 8290 6942 5868 5614 5194 4520 4378 4328 | 1.59 1.806 1.765 1.778 1.912 1.897 1.937 1.955 |
| LJMUCV267 | 8340 6814 5888 5646 5134 4498 4378 4318 | 1.397 1.565 1.549 1.562 1.669 1.663 1.682 1.694 |
| LJMUCV268 | 8168 6824 5902 5630 5168 4530 4400 4294 | 1.563 1.715 1.707 1.715 1.796 1.796 1.817 1.826 |
| LJMUCV269 | 8350 6850 5902 5652 5154 4510 4414 4310 | 1.617 1.731 1.725 1.734 1.815 1.816 1.833 1.846 |
| LJMUCV270 | 8316 6882 5844 5602 5170 4400 4324 | 1.469 1.696 1.631 1.647 1.867 1.785 1.804 |
| LJMUCV271 | 8210 6884 5888 5168 4524 4412 4312 | 1.501 1.644 1.627 1.698 1.699 1.709 1.716 |
| LJMUCV272 | 8276 6880 5886 5636 5178 4526 4408 4322 | 1.428 1.705 1.657 1.679 1.786 1.791 1.814 1.829 |
| LJMUCV273 | 8404 6894 5882 5598 5158 4406 4338 | 1.621 1.95 1.833 1.874 2.072 2.032 2.051 |
| LJMUCV274 | 8654 8352 6904 5874 5618 5168 4542 4396 4326 | 1.421 1.433 1.688 1.563 1.594 1.813 1.706 1.744 1.77 |
| LJMUCV275 | 9690 8024 6764 5834 5628 5168 4496 4408 4314 | 1.329 1.399 1.558 1.552 1.553 1.618 1.624 1.635 1.65 |
| LJMUCV276 | 9808 8134 6824 5866 5630 5168 4505 4336 | 1.139 1.352 1.786 1.742 1.76 1.848 1.85 1.873 |
| LJMUCV277 | 8252 6816 5878 5652 5150 4532 4382 4326 | 1.512 1.646 1.633 1.637 1.729 1.71 1.737 1.752 |
| LJMUCV278 | 9772 8068 6924 5836 5610 5144 | 0.959 1.376 2.04 1.986 2.007 2.136 |
| LJMUCV279 | 8362 8224 6870 5886 5636 5190 4532 4410 4326 | 1.325 1.332 1.65 1.577 1.601 1.742 1.717 1.739 1.758 |
| LJMUCV280 | 8366 6848 5916 5624 5178 4534 4398 4334 | 1.472 1.716 1.682 1.706 1.798 1.806 1.825 1.834 |
| LJMUCV281 | 8380 6934 5868 5650 5172 4492 4402 4312 | 1.471 1.558 1.567 1.572 1.636 1.653 1.669 1.679 |
| LJMUCV282 | 9758 8068 6780 5910 5638 5128 4528 4378 4312 | 1.222 1.381 1.639 1.634 1.637 1.695 1.716 1.729 1.738 |
| LJMUCV283 | 8414 6968 5884 5632 5152 4504 4380 4330 | 1.551 1.765 1.734 1.755 1.866 1.865 1.899 1.913 |
| LJMUCV284 | 8342 6938 6024 5866 5646 5154 4398 4334 | 1.622 1.804 1.756 1.78 1.792 1.914 1.945 1.959 |
| LJMUCV285 | 8532 8336 6902 5870 5760 5578 5182 4556 4406 4312 | 1.608 1.625 1.915 1.85 1.859 1.875 1.995 1.98 2.013 2.028 |
| LJMUCV287 | 8368 6908 5878 5646 5138 4508 4402 4328 4174 | 1.665 1.812 1.805 1.811 1.886 1.895 1.921 1.936 1.95 |
| LJMUCV288 | 8344 6888 5880 5634 5182 4540 4400 4324 | 1.238 1.344 1.325 1.334 1.449 1.401 1.419 1.429 |
| LJMUCV289 | 8096 6772 5616 5124 | 1.093 1.784 1.657 1.843 |
| LJMUCV290 | 8340 6906 5910 5630 5192 4524 4378 4316 | 1.429 1.674 1.604 1.635 1.748 1.736 1.761 1.772 |
| LJMUCV291 | 8186 6936 5886 5638 5180 4488 4410 4328 | 1.476 1.578 1.578 1.582 1.634 1.651 1.66 1.668 |
| LJMUCV292 | 8202 6882 5912 5630 5168 4520 4396 4312 | 1.529 1.692 1.662 1.672 1.771 1.758 1.775 1.786 |
| LJMUCV293 | 9666 7988 6914 5180 4526 | 0.902 1.225 1.514 1.589 1.578 |
| LJMUCV294 | 8690 8402 6878 5874 5638 5168 4534 4394 4318 | 1.516 1.545 1.874 1.769 1.797 2 1.945 1.985 2.004 |
| LJMUCV295 | 8382 6808 5672 5644 5156 4498 4402 4330 | 1.454 1.542 1.542 1.544 1.598 1.604 1.622 1.634 |
| LJMUCV296 | 9748 8060 6854 5888 5610 5182 4532 4388 4316 | 1.461 1.573 1.84 1.824 1.824 1.921 1.934 1.953 1.966 |
| LJMUCV297 | 8342 6976 5878 5652 5182 4492 4410 4330 | 1.573 1.664 1.659 1.664 1.708 1.709 1.722 1.728 |
| LJMUCV298 | 8322 7058 5906 5660 5180 4506 4394 4310 | 1.479 1.561 1.579 1.59 1.635 1.671 1.689 1.695 |
| LJMUCV345 | 8170 6910 6024 5600 5156 4530 4376 4308 | 1.398 1.593 1.552 1.575 1.671 1.669 1.696 1.71 |
| LJMUCV346 | 8406 6824 6012 5668 5184 4406 4338 | 1.345 1.426 1.421 1.432 1.481 1.5 1.506 |
| LJMUCV347 | 8608 8342 6852 5868 5624 5158 4532 4372 4310 | 1.331 1.349 1.549 1.51 1.524 1.655 1.636 1.669 1.685 |
| LJMUCV348 | 9826 8212 6882 5862 5618 5168 4514 4324 | 1.234 1.322 1.609 1.533 1.552 1.73 1.672 1.712 |
| LJMUCV349 | 8398 6868 5886 5640 5168 4520 4400 4318 | 1.482 1.718 1.646 1.671 1.795 1.769 1.799 1.818 |
| LJMUCV350 | 8608 8370 6906 5876 5632 5196 4520 4404 4318 | 1.349 1.357 1.484 1.451 1.466 1.543 1.534 1.551 1.563 |
| LJMUCV351 | 8664 8388 6940 5890 5624 5158 4526 4402 4330 | 1.516 1.547 1.913 1.808 1.847 2.029 1.989 2.029 2.049 |
| LJMUCV352 | 9784 8196 6822 6000 5578 5152 4522 4404 4314 | 1.259 1.336 1.568 1.523 1.543 1.638 1.639 1.658 1.673 |
| LJMUCV353 | 8626 8322 6930 5878 5640 5162 4520 4398 4326 | 1.711 1.729 1.885 1.886 1.897 1.969 1.987 2.023 2.038 |
| LJMUCV354 | 8374 6864 6040 5878 5640 5168 4516 4394 4328 | 1.589 1.731 1.712 1.728 1.734 1.832 1.822 1.848 1.86 |
| LJMUCV355 | 8640 8360 6892 5864 5592 5182 4530 4394 4310 | 1.383 1.401 1.779 1.604 1.655 1.905 1.815 1.839 1.858 |
| LJMUCV356 | 9756 8004 6818 5632 5248 4524 | 0.898 1.379 1.961 1.944 2.013 2.027 |
| LJMUCV357 | 8648 8354 6866 5872 5740 5610 5140 4526 4402 4298 | 1.395 1.419 1.724 1.642 1.653 1.669 1.823 1.794 1.822 1.841 |
| LJMUCV358 | 8668 8390 6912 5894 5762 5638 5156 4518 4400 4310 | 1.521 1.542 1.882 1.754 1.774 1.799 2.012 1.951 1.993 2.019 |
| LJMUCV359 | 8304 6906 5892 5610 5184 4514 4404 4314 | 1.389 1.675 1.605 1.637 1.744 1.736 1.754 1.768 |
| LJMUCV360 | 8354 6880 6010 5868 5656 5156 4656 4398 4332 | 1.678 1.857 1.829 1.849 1.857 1.943 1.919 1.986 2.002 |
| LJMUCV361 | 8666 8414 6920 5910 5608 5174 4520 4398 4306 | 1.383 1.397 1.522 1.503 1.514 1.578 1.576 1.596 1.603 |
| LJMUCV362 | 8676 8376 6972 6026 5870 5770 5656 5188 4518 4394 4302 | 1.466 1.483 1.695 1.605 1.62 1.628 1.639 1.777 1.751 1.785 1.801 |
| LJMUCV363 | 8208 6866 5898 5610 5142 4544 4390 4322 | 1.558 1.901 1.851 1.869 2.055 1.994 2.03 2.049 |
| LJMUCV364 | 8360 6910 5880 5630 5186 4392 4308 | 1.586 1.859 1.75 1.782 1.942 1.963 1.965 |
| LJMUCV365 | 8366 6914 5874 5634 5188 4540 4398 4316 | 1.561 1.889 1.779 1.819 1.997 1.949 1.989 2.009 |
| LJMUCV366 | 8296 6914 6014 5766 5646 5162 4514 4410 4338 | 1.696 1.844 1.821 1.836 1.838 1.906 1.911 1.932 1.941 |
| LJMUCV367 | 9748 8084 6842 5910 5610 5166 4518 4366 4306 | 1.265 1.437 1.805 1.783 1.786 1.899 1.911 1.936 1.951 |
| LJMUCV368 | 9732 8152 6902 5876 5626 5168 4498 4400 4316 | 1.147 1.213 1.34 1.352 1.358 1.413 1.439 1.454 1.465 |
| LJMUCV369 | 9748 8050 6918 5274 5626 4552 | 0.872 1.325 1.825 1.839 1.806 1.839 |
| LJMUCV370 | 8674 8244 6864 5608 5180 | 1.194 1.209 1.606 1.398 2.035 |
| LJMUCV371 | 8384 6892 5876 5612 5168 4516 4404 4312 | 1.489 1.742 1.678 1.703 1.819 1.801 1.825 1.839 |
| LJMUCV372 | 8388 6910 5866 5656 5150 4516 4378 4334 | 1.535 1.669 1.659 1.669 1.751 1.766 1.789 1.799 |
| LJMUCV373 | 8648 8332 6886 5920 5644 5168 4548 4414 4334 | 1.334 1.35 1.545 1.493 1.514 1.642 1.606 1.626 1.64 |
| LJMUCV374 | 9704 7988 6866 5182 4534 | 0.981 1.318 1.536 1.603 1.605 |
| LJMUCV375 | 8244 6858 6022 5648 5180 4400 4324 | 1.619 1.813 1.771 1.791 1.884 1.894 1.904 |
| LJMUCV376 | 8228 6842 5892 5626 5152 4504 4392 4322 | 1.556 1.784 1.76 1.769 1.881 1.884 1.909 1.923 |
| LJMUCV377 | 9714 7964 6842 5142 4544 4398 4310 | 1.112 1.453 1.707 1.805 1.774 1.795 1.815 |
| LJMUCV378 | 8094 6850 5870 5580 5148 4514 4406 4328 | 1.375 1.665 1.637 1.64 1.746 1.745 1.764 1.782 |
| LJMUCV379 | 8682 8324 6870 5878 5650 5184 4524 4400 4324 4220 | 1.466 1.491 1.735 1.677 1.699 1.829 1.803 1.835 1.849 1.861 |
| LJMUCV380 | 8392 6904 6026 5884 5682 5146 4356 4316 | 1.441 1.528 1.517 1.529 1.533 1.594 1.63 1.64 |
| LJMUCV381 | 8074 6910 5908 5644 5168 4514 4366 4320 | 1.716 1.93 1.929 1.938 2.031 2.035 2.064 2.077 |
| LJMUCV382 | 8252 6866 5876 5644 5158 4514 4380 4334 | 1.689 1.848 1.833 1.834 1.909 1.911 1.942 1.955 |
| LJMUCV383 | 8250 6906 5868 5635 5174 4526 4406 4330 | 1.359 1.765 1.644 1.672 1.876 1.821 1.851 1.876 |
| LJMUCV384 | 8294 6856 6018 5660 5624 5196 4408 4314 | 1.366 1.505 1.468 1.476 1.481 1.552 1.565 1.576 |
| LJMUCV385 | 8278 6842 5900 5650 5168 4546 4382 4324 | 1.345 1.508 1.475 1.488 1.616 1.579 1.604 1.617 |
| LJMUCV386 | 8308 6896 5900 5620 5204 4404 4314 | 1.287 1.506 1.441 1.466 1.601 1.587 1.606 |
| LJMUCV387 | 8254 6876 5878 5624 5138 4554 4404 4318 | 1.435 1.8 1.71 1.744 1.894 1.878 1.906 1.921 |
| LJMUCV388 | 8332 6892 5918 5632 5154 4538 4398 4326 | 1.563 1.929 1.824 1.858 2.048 1.997 2.044 2.069 |
| LJMUCV389 | 8164 6840 5868 5660 5182 4528 4400 4312 | 1.492 1.685 1.665 1.668 1.789 1.759 1.779 1.793 |
| LJMUCV390 | 8148 6868 6022 5658 5168 4394 4318 | 1.449 1.861 1.635 1.647 1.762 1.783 1.796 |
| LJMUCV391 | 8362 6904 5866 5616 5166 4550 4396 4324 | 1.458 1.789 1.688 1.724 1.903 1.852 1.888 1.906 |
| LJMUCV392 | 8310 6826 5904 5644 5160 4536 4394 4328 | 1.644 1.812 1.789 1.797 1.919 1.891 1.919 1.935 |
| LJMUCV393 | 8376 6904 5882 5664 5180 4390 4312 | 1.532 1.722 1.671 1.689 1.792 1.803 1.815 |
| LJMUCV394 | 8364 6908 5878 5622 5198 4508 4404 4318 | 1.502 1.651 1.622 1.634 1.743 1.715 1.737 1.75 |
| LJMUCV395 | 8398 6920 5904 5626 5194 4534 4386 4334 | 1.537 1.928 1.786 1.836 2.066 1.985 2.028 2.051 |
| LJMUCV396 | 8366 6854 5904 5636 5184 4522 4400 4330 | 1.627 1.838 1.786 1.806 1.969 1.903 1.938 1.956 |
| LJMUCV397 | 8304 6944 5874 5632 5194 4490 4316 | 1.304 1.644 1.589 1.619 1.693 1.693 1.712 |
| LJMUCV398 | 8388 6912 5876 5620 5162 4522 4396 4326 | 1.545 1.922 1.812 1.854 2.048 2.015 2.06 2.083 |
| LJMUCV399 | 8334 6896 5846 5612 5170 4542 4408 4312 | 1.483 1.723 1.664 1.692 1.812 1.799 1.823 1.839 |
| LJMUCV400 | 8570 8358 6882 5858 5638 5188 4402 4304 | 1.506 1.518 1.741 1.681 1.697 1.831 1.829 1.847 |
| LJMUCV401 | 8654 8394 6842 5892 5634 5182 4528 4406 4326 | 1.524 1.545 1.876 1.769 1.816 2.018 1.973 2.006 2.029 |
| LJMUCV402 | 8652 8406 6936 6010 5882 5630 5172 4394 4336 | 1.633 1.656 1.926 1.834 1.857 1.883 2.025 2.055 2.069 |
| LJMUCV403 | 8388 6942 5896 5642 5166 4530 4404 4320 | 1.548 1.815 1.734 1.768 1.924 1.899 1.936 1.958 |
| LJMUCV404 | 8632 6942 5890 5644 5182 4518 4406 4324 | 1.551 1.807 1.734 1.766 1.912 1.899 1.929 1.949 |
| LJMUCV405 | 8642 8396 6900 5900 5578 5158 4398 4300 | 1.465 1.482 1.837 1.669 1.721 2.026 1.94 1.981 |
| LJMUCV406 | 8350 6844 5602 5158 | 1.208 1.603 1.398 2.033 |
| LJMUCV407 | 8344 6894 5860 5612 5168 | 1.457 1.809 1.669 1.714 1.911 |
| LJMUCV408 | 8646 8354 6918 5862 5624 5168 4402 4330 | 1.3 1.315 1.505 1.448 1.465 1.583 1.578 1.59 |
| LJMUCV409 | 8636 8362 6942 5872 5656 5180 4492 4408 4312 | 1.599 1.614 1.825 1.774 1.788 1.924 1.909 1.942 1.961 |
| LJMUCV410 | 8110 6850 5898 5586 5144 | 1.574 2.009 1.972 2.002 2.088 |
| LJMUCV411 | 8670 8376 6906 5886 5604 5182 4536 4392 4310 | 1.463 1.481 1.735 1.64 1.672 1.869 1.779 1.804 1.822 |
| LJMUCV412 | 8680 8366 6878 5884 5596 5206 4524 4400 4316 | 1.586 1.608 1.797 1.77 1.782 1.884 1.871 1.911 1.931 |
| LJMUCV413 | 8662 8374 6928 5882 5634 5172 4498 4396 4326 | 1.535 1.552 1.798 1.718 1.749 1.9 1.887 1.925 1.944 |
| LJMUCV414 | 8654 6906 5864 5612 5162 4498 4412 4318 | 1.56 1.86 1.788 1.819 1.968 1.956 1.983 2.007 |
| LJMUCV415 | 8668 8346 6912 5866 5582 5182 4522 4398 4306 | 1.391 1.416 1.722 1.623 1.661 1.819 1.782 1.805 1.824 |
| LJMUCV416 | 8364 6904 5880 5620 5190 4516 4398 4322 | 1.427 1.653 1.591 1.616 1.729 1.716 1.744 1.759 |
| LJMUCV417 | 8660 8352 6906 5578 5204 | 1.209 1.222 1.715 1.504 1.883 |
| LJMUCV418 | 8258 6832 5624 5210 4380 4310 | 1.519 2.102 2.032 2.171 2.199 2.216 |
| LJMUCV419 | 8278 6944 5918 5642 5176 4396 4278 | 1.672 1.987 1.884 1.925 2.122 2.119 2.142 |
| LJMUCV420 | 8364 6890 5604 5168 | 1.477 2.231 2.001 2.416 |
| LJMUCV421 | 8682 8362 6898 5910 5632 5166 4498 4400 4314 | 1.437 1.453 1.687 1.615 1.644 1.805 1.77 1.816 1.84 |
| LJMUCV422 | 8682 8386 6930 6008 5632 5198 4408 4330 4324 | 1.622 1.647 1.949 1.826 1.875 2.064 2.058 2.089 2.099 |
| LJMUCV423 | 8378 6906 6040 5644 5188 4400 4340 | 1.546 1.736 1.674 1.706 1.845 1.848 1.859 |
| LJMUCV424 | 8680 8408 6936 6024 5638 5170 4408 4328 | 1.438 1.449 1.577 1.548 1.577 1.702 1.713 1.73 |
| LJMUCV425 | 8638 8348 6866 5914 5632 5156 4504 4398 4304 | 1.431 1.444 1.638 1.569 1.594 1.752 1.698 1.72 1.739 |
| LJMUCV426 | 8208 6814 6016 5628 5168 4532 4400 4308 | 1.335 1.479 1.456 1.469 1.588 1.557 1.578 1.595 |
| LJMUCV427 | 8122 6788 6012 5650 5152 4512 4398 4312 | 1.119 1.216 1.206 1.216 1.299 1.292 1.304 1.315 |
| LJMUCV428 | 8344 6894 5874 5638 5160 4520 4374 4340 | 1.534 1.674 1.657 1.668 1.749 1.751 1.775 1.783 |
| LJMUCV429 | 8702 8332 6032 5868 5622 5170 4396 4310 | 1.522 1.537 1.635 1.65 1.664 1.819 1.798 1.816 |
| LJMUCV430 | 9750 8016 6814 5578 5112 4534 | 0.811 1.231 1.675 1.644 1.708 1.678 |
| LJMUCV431 | 8626 8388 7024 5904 5620 5080 4528 | 0.744 0.757 1.939 0.901 0.926 0.967 0.973 |
| LJMUCV432 | 9772 7988 6840 5582 5114 4536 | 0.866 1.295 1.785 1.752 1.816 1.788 |
| LJMUCV433 | 8342 6828 5910 5618 5184 4536 4382 4314 | 1.596 1.804 1.768 1.779 1.915 1.869 1.897 1.913 |
| LJMUCV434 | 8352 6912 5868 5636 5182 4394 4334 | 1.431 1.688 1.587 1.607 1.891 1.769 1.792 |
| LJMUCV435 | 8616 8404 6906 5864 5642 5196 4398 4312 4238 | 1.677 1.694 2.131 1.932 1.977 2.319 2.218 2.26 2.289 |
| LJMUCV437 | 8630 8264 6806 5900 5586 5186 4564 4398 4312 | 1.597 1.618 1.825 1.794 1.805 1.933 1.895 1.931 1.953 |
| LJMUCV438 | 8674 8348 6862 5882 5620 5168 4538 4410 4320 | 1.432 1.453 1.727 1.617 1.65 1.831 1.769 1.799 1.826 |
| LJMUCV439 | 8640 8362 6908 5900 5606 5204 4518 4392 4288 | 1.472 1.488 1.699 1.637 1.662 1.763 1.747 1.773 1.785 |
| LJMUCV440 | 8164 6828 5902 5612 5168 4524 4390 4320 | 1.509 1.763 1.726 1.734 1.979 1.852 1.879 1.899 |
| LJMUCV441 | 8300 6910 5884 5624 5172 4398 4310 | 1.379 1.613 1.54 1.565 1.695 1.684 1.698 |
| LJMUCV442 | 8252 6802 5870 5610 5160 4518 4400 4326 | 1.456 1.661 1.621 1.629 1.792 1.742 1.779 1.803 |
| LJMUCV443 | 9676 8078 6828 5910 5184 4538 4296 | 1.37 1.476 1.677 1.678 1.749 1.754 1.776 |
| LJMUCV444 | 8204 6822 5878 5624 5144 4544 4396 4328 | 1.485 1.741 1.687 1.689 1.853 1.796 1.821 1.845 |
| LJMUCV445 | 8358 6894 5878 5632 5206 4548 4392 4318 | 1.486 1.792 1.687 1.722 1.912 1.847 1.882 1.901 |
| LJMUCV446 | 8692 8374 6912 5908 5614 5210 4526 4392 4318 | 1.456 1.474 1.727 1.637 1.673 1.881 1.794 1.828 1.848 |
| LJMUCV447 | 8674 8406 6950 5880 5656 5188 4404 4326 4224 | 1.589 1.61 1.963 1.812 1.849 2.085 2.037 2.069 2.084 |
| LJMUCV448 | 8624 8336 6892 5892 5604 5172 4542 4394 4310 | 1.371 1.38 1.538 1.489 1.509 1.641 1.595 1.622 1.636 |
| LJMUCV449 | 8364 6938 5888 5596 5190 4524 4394 4314 | 1.544 1.767 1.678 1.706 1.896 1.833 1.872 1.895 |
| LJMUCV450 | 8626 8362 6892 5864 5610 5180 4530 4384 4310 | 1.524 1.554 1.907 1.818 1.855 2.015 2.005 2.035 2.051 |
| LJMUCV451 | 8620 6910 5620 5188 4502 | 1.114 1.307 1.227 1.644 1.322 |
| LJMUCV452 | 8678 8416 6952 5870 5634 5204 4520 4390 4318 | 1.602 1.623 1.829 1.778 1.797 1.957 1.908 1.941 1.959 |
| LJMUCV453 | 8350 6934 5888 5580 5184 4524 4402 4324 | 1.549 1.689 1.652 1.668 1.764 1.744 1.771 1.786 |
| LJMUCV454 | 8690 8376 6900 5912 5630 5158 4528 4370 4328 | 1.438 1.462 1.703 1.633 1.661 1.795 1.768 1.799 1.512 |
| LJMUCV455 | 8386 6932 5878 5624 5158 4372 4532 4330 | 1.463 1.701 1.638 1.661 1.795 1.797 1.768 1.811 |
| LJMUCV456 | 8644 8422 6930 5876 5624 5156 4540 4416 4328 | 1.464 1.482 1.809 1.698 1.738 1.922 1.875 1.929 1.905 |
| LJMUCV457 | 8694 8364 6936 5878 5626 5166 4520 4404 4308 | 1.556 1.587 1.91 1.822 1.857 2.012 1.989 2.012 2.035 |
| LJMUCV458 | 8658 8254 6864 6016 5650 5166 4492 4400 4320 | 1.262 1.277 1.405 1.379 1.404 1.491 1.5 1.515 1.525 |
