# Supplementary material for: Evaluation of portable near-infrared spectroscopy for authentication of mRNA based COVID-19 vaccines
Source: PLoS One. 2022 May 4;17(5):e0267214. doi: 10.1371/journal.pone.0267214 (PMC9067670; doi:10.1371/journal.pone.0267214)
Supplement: S2 Table — (DOCX) [file pone.0267214.s002.docx]

S2 table. Spectral quality of the vaccines studied

| LJMUCV | N | Max peak  position (cm^-1^) | Max peak intensity (absorbance units) | SNR |
| --- | --- | --- | --- | --- |
| LJMUCV1 | 9 | 4318 | 2.10 | 30.5 |
| LMUCV2 | 4 | 5172 | 2.63 | 19.8 |
| LMUCV3 | 9 | 4336 | 2.08 | 46.8 |
| LJMUCV4 | 5 | 5146 | 2.72 | 165 |
| LJMUCV5 | 9 | 4332 | 1.78 | 19.5 |
| LJMUCV6 | 9 | 5166 | 1.89 | 17.3 |
| LJMUCV7 | 9 | 4292 | 1.85 | 76.3 |
| LJMUCV8 | 4 | 5182 | 2.66 | 20.2 |
| LJMUCV9 | 9 | 4332 | 1.95 | 36.5 |
| LJMUCV10 | 8 | 4324 | 1.86 | 27.3 |
| LJMUCV11 | 10 | 4324 | 1.86 | 27.3 |
| LJMUCV12 | 7 | 5192 | 1.70 | 19 |
| LJMUCV13 | 5 | 5174 | 2.52 | 46.7 |
| LJMUCV14 | 5 | 5150 | 2.51 | 5.92 |
| LJMUCV15 | 4 | 5156 | 1.80 | 11.8 |
| LJMUCV16 | 8 | 4272 | 2.02 | 23 |
| LJMUCV17 | 4 | 5174 | 1.99 | 5.77 |
| LJMUCV18 | 4 | 5152 | 2.15 | 6.96 |
| LJMUCV19 | 8 | 4334 | 1.94 | 19 |
| LJMUCV20 | 9 | 4238 | 1.75 | 8.11 |
| LJMUCV21 | 7 | 5180 | 1.84 | 18.9 |
| LJMUCV22 | 7 | 5168 | 2.18 | 122 |
| LJMUCV23 | 9 | 4324 | 1.95 | 22.4 |
| LJMUCV24 | 6 | 5202 | 2.12 | 29.6 |
| LJMUCV25 | 3 | 5192 | 1.72 | 71.6 |
| LJMUCV26 | 5 | 5196 | 2.12 | 74 |
| LJMUCV27 | 9 | 5190 | 2.08 | 77.9 |
| LJMUCV28 | 7 | 5210 | 2.07 | 21.2 |
| LJMUCV29 | 4 | 5184 | 2.28 | 8.06 |
| LJMUCV30 | 9 | 5174 | 1.87 | 55.3 |
| LJMUCV31 | 10 | 4182 | 1.84 | 12.3 |
| LJMUCV32 | 9 | 4310 | 2.00 | 18 |
| LJMUCV33 | 6 | 5170 | 1.78 | 75 |
| LJMUCV34 | 7 | 4332 | 2.04 | 70.1 |
| LJMUCV35 | 8 | 4292 | 2.06 | 33.8 |
| LJMUCV36 | 5 | 5178 | 2.36 | 44.9 |
| LJMUCV37 | 8 | 4338 | 1.99 | 103 |
| LJMUCV38 | 4 | 6840 | 2.48 | 26.8 |
| LJMUCV39 | 4 | 5194 | 2.57 | 20.4 |
| LJMUCV40 | 5 | 5092 | 2.66 | 18.9 |
| LJMUCV41 | 4 | 5166 | 2.57 | 25.4 |
| LJMUCV42 | 8 | 4312 | 2.06 | 21.3 |
| LJMUCV43 | 9 | 4302 | 2.03 | 80.9 |
| LJMUCV44 | 9 | 4326 | 2.00 | 42.6 |
| LJMUCV45 | 9 | 5178 | 1.89 | 60.8 |
| LJMUCV46 | 8 | 4328 | 1.98 | 36.7 |
| LJMUCV47 | 9 | 5168 | 2.05 | 44.9 |
| LJMUCV48 | 8 | 5182 | 2.14 | 48.9 |
| LJMUCV49 | 9 | 4314 | 1.92 | 40.5 |
| LJMUCV50 | 9 | 5196 | 1.84 | 17.8 |
| LJMUCV51 | 8 | 4324 | 1.64 | 20.7 |
| LJMUCV52 | 5 | 4522 | 1.75 | 9.2 |
| LJMUCV53 | 9 | 4280 | 1.81 | 13.5 |
| LJMUCV54 | 8 | 4306 | 1.63 | 13.7 |
| LJMUCV55 | 6 | 5188 | 1.75 | 7.4 |
| LJMUCV56 | 9 | 4308 | 1.76 | 13.7 |
| LJMUCV57 | 9 | 4312 | 1.97 | 32.7 |
| LJMUCV58 | 9 | 5160 | 1.82 | 7.4 |
| LJMUCV59 | 9 | 4332 | 1.79 | 28.7 |
| LJMUCV60 | 7 | 4326 | 1.85 | 14.1 |
| LJMUCV61 | 7 | 4326 | 1.79 | 11.6 |
| LJMUCV62 | 9 | 4332 | 1.79 | 15.5 |
| LJMUCV63 | 5 | 4544 | 1.75 | 26.2 |
| LJMUCV64 | 8 | 4302 | 1.81 | 16.9 |
| LJMUCV65 | 8 | 5170 | 1.72 | 10.3 |
| LJMUCV66 | 6 | 5146 | 1.75 | 24.7 |
| LJMUCV67 | 8 | 5158 | 1.84 | 54.5 |
| LJMUCV68 | 8 | 4320 | 1.61 | 25 |
| LJMUCV69 | 8 | 5154 | 1.71 | 12.8 |
| LJMUCV70 | 9 | 4318 | 2.04 | 55.5 |
| LJMUCV71 | 9 | 5192 | 1.82 | 58.7 |
| LJMUCV72 | 9 | 5182 | 1.87 | 10.7 |
| LJMUCV73 | 9 | 5176 | 2.03 | 175 |
| LJMUCV74 | 9 | 4338 | 2.05 | 189 |
| LJMUCV75 | 10 | 5196 | 1.87 | 36.7 |
| LJMUCV76 | 6 | 5168 | 1.97 | 10.9 |
| LJMUCV77 | 4 | 5148 | 2.26 | 30.4 |
| LJMUCV78 | 9 | 4328 | 1.94 | 28.2 |
| LJMUCV79 | 8 | 5196 | 1.84 | 84.5 |
| LJMUCV80 | 9 | 5168 | 1.86 | 129 |
| LJMUCV81 | 7 | 5166 | 1.82 | 120 |
| LJMUCV82 | 9 | 5152 | 1.83 | 34.7 |
| LJMUCV83 | 8 | 4328 | 1.70 | 8.22 |
| LJMUCV84 | 9 | 5168 | 1.89 | 19.3 |
| LJMUCV85 | 9 | 5206 | 1.90 | 19.1 |
| LJMUCV86 | 9 | 5190 | 1.88 | 14.8 |
| LJMUCV87 | 8 | 5184 | 1.92 | 22.3 |
| LJMUCV88 | 9 | 5200 | 1.86 | 15.7 |
| LJMUCV89 | 9 | 4334 | 1.92 | 70.8 |
| LJMUCV90 | 9 | 5200 | 1.72 | 61.9 |
| LJMUCV91 | 9 | 4324 | 1.89 | 35.9 |
| LJMUCV92 | 9 | 4312 | 1.78 | 34.3 |
| LJMUCV93 | 9 | 4322 | 1.86 | 45.7 |
| LJMUCV94 | 9 | 4322 | 1.93 | 19.5 |
| LJMUCV95 | 9 | 5180 | 1.92 | 18.6 |
| LJMUCV96 | 9 | 5188 | 1.92 | 10.5 |
| LJMUCV97 | 9 | 5196 | 1.90 | 14.4 |
| LJMUCV98 | 9 | 5168 | 1.90 | 16.2 |
| LJMUCV99 | 9 | 4308 | 1.96 | 115 |
| LJMUCV100 | 5 | 4494 | 1.86 | 10.3 |
| LJMUCV101 | 9 | 4308 | 1.86 | 68.1 |
| LJMUCV102 | 11 | 4166 | 1.88 | 282 |
| LJMUCV103 | 9 | 5166 | 1.67 | 143 |
| LJMUCV104 | 5 | 5182 | 2.43 | 40.9 |
| LJMUCV105 | 10 | 4248 | 1.99 | 53.2 |
| LJMUCV106 | 9 | 5206 | 1.79 | 41.1 |
| LJMUCV107 | 9 | 5166 | 1.53 | 23.7 |
| LJMUCV108 | 9 | 5178 | 1.90 | 122 |
| LJMUCV109 | 9 | 5166 | 1.89 | 23.3 |
| LJMUCV110 | 7 | 5176 | 1.59 | 22.8 |
| LJMUCV111 | 10 | 5186 | 2.00 | 23.3 |
| LJMUCV112 | 9 | 5168 | 1.93 | 38.7 |
| LJMUCV113 | 9 | 5204 | 1.97 | 50.9 |
| LJMUCV114 | 9 | 5168 | 1.93 | 19.9 |
| LJMUCV115 | 9 | 5182 | 1.90 | 86.6 |
| LJMUCV116 | 9 | 5168 | 1.75 | 15.5 |
| LJMUCV117 | 9 | 5198 | 1.90 | 22.8 |
| LJMUCV118 | 9 | 5180 | 1.72 | 30.4 |
| LJMUCV119 | 8 | 5152 | 1.88 | 15.4 |
| LJMUCV120 | 7 | 5170 | 1.82 | 26.1 |
| LJMUCV121 | 8 | 5154 | 1.85 | 31.7 |
| LJMUCV122 | 8 | 4298 | 1.56 | 24.1 |
| LJMUCV123 | 8 | 4310 | 1.58 | 30.6 |
| LJMUCV124 | 8 | 4328 | 1.80 | 128 |
| LJMUCV125 | 8 | 4290 | 1.72 | 26.6 |
| LJMUCV126 | 8 | 4320 | 1.47 | 70.6 |
| LJMUCV127 | 9 | 4232 | 1.83 | 24 |
| LJMUCV128 | 8 | 4324 | 1.90 | 13 |
| LJMUCV129 | 9 | 5154 | 1.67 | 75 |
| LJMUCV130 | 8 | 5168 | 1.67 | 31 |
| LJMUCV131 | 7 | 4308 | 1.62 | 50.6 |
| LJMUCV132 | 7 | 5166 | 1.64 | 30.3 |
| LJMUCV133 | 7 | 4224 | 1.64 | 16.2 |
| LJMUCV134 | 8 | 4302 | 1.79 | 10.4 |
| LJMUCV135 | 7 | 5168 | 1.55 | 34.7 |
| LJMUCV136 | 8 | 5174 | 2.08 | 289 |
| LJMUCV137 | 8 | 4332 | 1.84 | 56.9 |
| LJMUCV138 | 8 | 4312 | 1.54 | 33.4 |
| LJMUCV139 | 5 | 5214 | 1.76 | 13.5 |
| LJMUCV140 | 8 | 4314 | 1.60 | 33.3 |
| LJMUCV141 | 8 | 4318 | 1.50 | 34.3 |
| LJMUCV142 | 8 | 5142 | 1.92 | 8.85 |
| LJMUCV143 | 9 | 5170 | 1.76 | 28.1 |
| LJMUCV144 | 8 | 4330 | 1.92 | 24.1 |
| LJMUCV145 | 8 | 4310 | 1.66 | 28.9 |
| LJMUCV146 | 8 | 4310 | 1.66 | 39.7 |
| LJMUCV147 | 8 | 5156 | 1.77 | 17.4 |
| LJMUCV148 | 6 | 4508 | 1.70 | 7.39 |
| LJMUCV149 | 8 | 4324 | 2.05 | 28.3 |
| LJMUCV150 | 8 | 5154 | 1.60 | 23.9 |
| LJMUCV151 | 8 | 4312 | 1.93 | 34 |
| LJMUCV152 | 8 | 4328 | 2.00 | 10.6 |
| LJMUCV153 | 8 | 4318 | 1.62 | 11.0 |
| LJMUCV154 | 9 | 4316 | 1.92 | 24.3 |
| LJMUCV155 | 8 | 4328 | 1.85 | 17.4 |
| LJMUCV156 | 8 | 5146 | 2.08 | 19.1 |
| LMUCV157 | 8 | 4278 | 1.77 | 22.9 |
| LJMUCV158 | 8 | 4294 | 1.50 | 25.4 |
| LJMUCV159 | 8 | 5154 | 1.87 | 28.8 |
| LJMUCV160 | 7 | 4322 | 1.74 | 27.4 |
| LJMUCV161 | 8 | 4240 | 1.84 | 14.7 |
| LJMUCV162 | 9 | 4172 | 1.98 | 26.0 |
| LJMUCV163 | 11 | 4204 | 1.80 | 31.2 |
| LJMUCV164 | 8 | 4306 | 1.72 | 16.9 |
| LJMUCV165 | 9 | 4310 | 1.63 | 17.2 |
| LJMUCV166 | 7 | 4286 | 2.19 | 7.49 |
| LJMUCV167 | 9 | 4298 | 1.55 | 18.7 |
| LJMUCV168 | 8 | 4532 | 1.94 | 30.1 |
| LJMUCV169 | 9 | 4334 | 1.81 | 14.5 |
| LJMUCV170 | 9 | 4288 | 1.51 | 18.0 |
| LJMUCV171 | 8 | 4156 | 1.92 | 22.4 |
| LJMUCV172 | 8 | 4322 | 2.05 | 22.4 |
| LJMUCV173 | 8 | 4328 | 2.06 | 32.2 |
| LJMUCV174 | 8 | 4320 | 1.84 | 13.8 |
| LJMUCV175 | 4 | 5164 | 1.98 | 5.53 |
| LJMUCV176 | 7 | 4326 | 1.99 | 16.7 |
| LJMUCV177 | 7 | 4304 | 1.84 | 18.4 |
| LJMUCV178 | 8 | 4312 | 1.67 | 21.7 |
| LJMUCV179 | 8 | 5182 | 1.86 | 20.1 |
| LJMUCV180 | 7 | 4272 | 1.85 | 53.8 |
| LJMUCV188 | 5 | 4496 | 1.89 | 24.9 |
| LJMUCV189 | 8 | 5182 | 1.58 | 72.1 |
| LJMUCV190 | 8 | 4306 | 1.86 | 52.7 |
| LJMUCV191 | 8 | 4316 | 1.80 | 27.2 |
| LJMUCV192 | 9 | 4330 | 1.85 | 26.7 |
| LJMUCV193 | 7 | 4310 | 1.74 | 64.4 |
| LJMUCV194 | 8 | 4312 | 1.96 | 22.8 |
| LJMUCV195 | 7 | 4328 | 2.10 | 17.9 |
| LJMUCV196 | 8 | 4320 | 1.85 | 26.8 |
| LJMUCV197 | 8 | 4330 | 1.79 | 16.7 |
| LJMUCV198 | 8 | 4312 | 1.94 | 11.5 |
| LJMUCV199 | 10 | 4318 | 1.73 | 42.4 |
| LJMUCV200 | 8 | 4310 | 1.89 | 12.7 |
| LJMUCV201 | 7 | 4332 | 1.81 | 43.6 |
| LJMUCV202 | 8 | 4300 | 1.73 | 33.6 |
| LJMUCV203 | 5 | 5154 | 1.81 | 19.7 |
| LJMUCV204 | 9 | 4304 | 1.52 | 24.2 |
| LJMUCV205 | 9 | 4330 | 1.65 | 12.5 |
| LJMUCV206 | 8 | 4288 | 1.66 | 15.4 |
| LJMUCV207 | 7 | 4318 | 1.95 | 13.6 |
| LJMUCV208 | 8 | 4320 | 1.65 | 28.5 |
| LJMUCV209 | 8 | 4324 | 1.97 | 43.6 |
| LJMUCV210 | 8 | 4318 | 1.93 | 50.9 |
| LJMUCV211 | 8 | 4322 | 2.08 | 36.6 |
| LJMUCV212 | 5 | 5208 | 2.01 | 21.4 |
| LJMUCV213 | 8 | 4336 | 1.79 | 11.9 |
| LJMUCV214 | 8 | 5182 | 1.79 | 12.4 |
| LJMUCV215 | 8 | 4328 | 1.67 | 24.1 |
| LJMUCV216 | 8 | 4294 | 1.92 | 45.3 |
| LJMUCV217 | 8 | 5168 | 1.87 | 17.3 |
| LJMUCV218 | 8 | 5214 | 1.81 | 48.0 |
| LJMUCV219 | 4 | 5188 | 1.69 | 19.4 |
| LJMUCV220 | 8 | 4320 | 1.90 | 7.12 |
| LJMUCV221 | 8 | 4306 | 2.08 | 69.6 |
| LJMUCV222 | 8 | 4296 | 1.71 | 28.1 |
| LJMUCV223 | 8 | 4334 | 1.66 | 19.2 |
| LJMUCV224 | 8 | 4274 | 1.61 | 8.20 |
| LJMUCV225 | 8 | 4330 | 1.64 | 8.30 |
| LJMUCV226 | 8 | 4326 | 1.96 | 15.9 |
| LJMUCV227 | 8 | 4326 | 1.99 | 47.9 |
| LJMUCV228 | 8 | 4324 | 1.65 | 31.6 |
| LJMUCV229 | 8 | 4326 | 1.85 | 12.8 |
| LJMUCV230 | 9 | 4336 | 2.03 | 17.9 |
| LJMUCV231 | 10 | 4326 | 1.73 | 33.4 |
| LJMUCV232 | 8 | 5172 | 1.63 | 9.48 |
| LJMUCV233 | 9 | 4302 | 1.73 | 37.7 |
| LJMUCV234 | 8 | 4310 | 1.48 | 42.4 |
| LJMUCV235 | 9 | 4312 | 1.89 | 91.8 |
| LJMUCV236 | 9 | 4298 | 1.67 | 35.9 |
| LJMUCV237 | 9 | 4528 | 1.71 | 21.0 |
| LJMUCV238 | 9 | 4312 | 1.79 | 32.4 |
| LJMUCV239 | 9 | 4326 | 1.88 | 7.02 |
| LJMUCV240 | 9 | 4326 | 1.83 | 57.7 |
| LJMUCV241 | 8 | 5168 | 1.94 | 52.8 |
| LJMUCV242 | 9 | 4310 | 1.63 | 20.8 |
| LJMUCV243 | 8 | 4328 | 1.83 | 18.2 |
| LJMUCV244 | 8 | 4314 | 2.00 | 18.2 |
| LJMUCV245 | 7 | 4316 | 1.95 | 24.4 |
| LJMUCV246 | 8 | 4336 | 1.74 | 11.7 |
| LJMUCV247 | 9 | 4310 | 1.90 | 42.9 |
| LJMUCV248 | 7 | 5266 | 2.09 | 26.3 |
| LJMUCV249 | 8 | 4320 | 1.69 | 35.4 |
| LJMUCV436 | 8 | 4326 | 1.90 | 10.5 |
| LJMUCV286 | 8 | 4332 | 1.87 | 10.5 |
| LJMUCV250 | 8 | 4320 | 1.51 | 41.1 |
| LJMUCV251 | 9 | 4330 | 1.55 | 14.8 |
| LJMUCV252 | 7 | 4310 | 1.51 | 38.7 |
| LJMUCV253 | 7 | 4322 | 1.97 | 11.8 |
| LJMUCV254 | 8 | 4318 | 2.01 | 16.3 |
| LJMUCV255 | 8 | 4318 | 1.85 | 24.4 |
| LJMUCV256 | 8 | 4298 | 1.76 | 12.1 |
| LJMUCV257 | 8 | 4336 | 1.57 | 17.0 |
| LJMUCV258 | 8 | 4290 | 1.81 | 78.1 |
| LJMUCV259 | 8 | 4324 | 1.71 | 12.8 |
| LJMUCV260 | 8 | 4314 | 1.85 | 133 |
| LJMUCV261 | 9 | 4304 | 1.84 | 50.8 |
| LJMUCV262 | 8 | 4316 | 1.64 | 14.4 |
| LJMUCV263 | 8 | 4334 | 1.62 | 22.0 |
| LJMUCV264 | 9 | 4324 | 2.00 | 18.3 |
| LJMUCV265 | 8 | 4328 | 1.96 | 18.0 |
| LJMUCV266 | 8 | 4318 | 1.69 | 64.2 |
| LJMUCV267 | 7 | 4294 | 1.83 | 20.5 |
| LJMUCV268 | 8 | 4310 | 1.85 | 23.1 |
| LJMUCV269 | 7 | 5170 | 1.87 | 42.5 |
| LJMUCV270 | 7 | 4312 | 1.72 | 23.3 |
| LJMUCV271 | 8 | 4322 | 1.83 | 14.4 |
| LJMUCV272 | 7 | 5158 | 2.07 | 22.4 |
| LJMUCV273 | 9 | 5168 | 1.81 | 16.9 |
| LJMUCV274 | 9 | 4314 | 1.65 | 40.6 |
| LJMUCV275 | 8 | 4336 | 1.87 | 17.6 |
| LJMUCV276 | 8 | 4326 | 1.75 | 13.0 |
| LJMUCV277 | 6 | 5144 | 2.14 | 46.4 |
| LJMUCV278 | 9 | 4326 | 1.76 | 7.46 |
| LJMUCV279 | 8 | 4334 | 1.83 | 14.7 |
| LJMUCV280 | 8 | 4312 | 1.68 | 22.6 |
| LJMUCV281 | 9 | 4312 | 1.74 | 42.9 |
| LJMUCV282 | 8 | 4330 | 1.91 | 23.5 |
| LJMUCV283 | 8 | 4334 | 1.96 | 87.9 |
| LJMUCV284 | 10 | 4312 | 2.03 | 101 |
| LJMUCV285 | 9 | 4174 | 1.95 | 9.77 |
| LJMUCV287 | 8 | 5182 | 1.45 | 34.5 |
| LJMUCV288 | 4 | 5124 | 1.84 | 61.4 |
| LJMUCV289 | 8 | 4316 | 1.77 | 5.10 |
| LJMUCV290 | 8 | 4328 | 1.67 | 11.5 |
| LJMUCV291 | 7 | 4312 | 1.79 | 11.1 |
| LJMUCV292 | 5 | 5180 | 1.59 | 32.9 |
| LJMUCV293 | 9 | 5168 | 2.00 | 16.9 |
| LJMUCV294 | 8 | 4330 | 1.63 | 17.3 |
| LJMUCV295 | 9 | 4316 | 1.97 | 31.4 |
| LJMUCV296 | 8 | 4330 | 1.73 | 16.9 |
| LJMUCV297 | 8 | 4310 | 1.70 | 189 |
| LJMUCV298 | 8 | 4308 | 1.71 | 39.8 |
| LJMUCV345 | 7 | 4338 | 1.51 | 12.4 |
| LJMUCV346 | 9 | 4310 | 1.69 | 15.8 |
| LJMUCV347 | 8 | 5168 | 1.73 | 24.2 |
| LJMUCV348 | 8 | 4318 | 1.82 | 13.5 |
| LJMUCV349 | 9 | 4318 | 1.56 | 16.9 |
| LJMUCV350 | 9 | 4330 | 2.05 | 45.1 |
| LJMUCV351 | 9 | 4314 | 1.67 | 17.0 |
| LJMUCV352 | 9 | 4326 | 2.04 | 13.8 |
| LJMUCV353 | 9 | 4328 | 1.86 | 62.1 |
| LJMUCV354 | 9 | 5182 | 1.91 | 43.1 |
| LJMUCV355 | 6 | 4524 | 2.03 | 13.2 |
| LJMUCV356 | 10 | 4298 | 1.84 | 5.68 |
| LJMUCV357 | 10 | 4310 | 2.02 | 31.8 |
| LJMUCV358 | 8 | 4314 | 1.77 | 120 |
| LJMUCV359 | 9 | 4332 | 2.00 | 55.5 |
| LJMUCV360 | 9 | 4306 | 1.60 | 42.8 |
| LJMUCV361 | 11 | 4302 | 1.80 | 21.3 |
| LJMUCV362 | 8 | 5142 | 2.06 | 41.9 |
| LJMUCV363 | 7 | 4308 | 1.97 | 26.0 |
| LJMUCV364 | 8 | 4316 | 2.01 | 19.4 |
| LJMUCV365 | 9 | 4338 | 1.94 | 57.9 |
| LJMUCV366 | 9 | 4306 | 1.95 | 32.9 |
| LJMUCV367 | 9 | 4316 | 1.47 | 17.5 |
| LJMUCV368 | 6 | 4552 | 1.84 | 14.4 |
| LJMUCV369 | 5 | 5180 | 2.04 | 17.9 |
| LJMUCV370 | 8 | 4312 | 1.84 | 10.1 |
| LJMUCV371 | 8 | 4334 | 1.80 | 49.7 |
| LJMUCV372 | 9 | 5168 | 1.64 | 48.6 |
| LJMUCV373 | 5 | 4534 | 1.61 | 29.3 |
| LJMUCV374 | 7 | 4324 | 1.90 | 36.2 |
| LJMUCV375 | 8 | 4322 | 1.92 | 37.1 |
| LJMUCV376 | 8 | 4310 | 1.82 | 36.2 |
| LJMUCV377 | 8 | 4328 | 1.78 | 29.7 |
| LJMUCV378 | 10 | 4220 | 1.86 | 12.0 |
| LJMUCV379 | 8 | 4316 | 1.64 | 31.7 |
| LJMUCV380 | 8 | 4320 | 2.08 | 18.6 |
| LJMUCV381 | 8 | 4334 | 1.96 | 16.9 |
| LJMUCV382 | 8 | 5174 | 1.88 | 33.3 |
| LJMUCV383 | 8 | 4314 | 1.58 | 17.7 |
| LJMUCV384 | 8 | 4324 | 1.62 | 9.04 |
| LJMUCV385 | 7 | 4314 | 1.61 | 18.1 |
| LJMUCV386 | 8 | 4318 | 1.92 | 32.4 |
| LJMUCV387 | 8 | 4326 | 2.07 | 14.4 |
| LJMUCV388 | 8 | 4312 | 1.79 | 29.4 |
| LJMUCV389 | 7 | 4318 | 1.80 | 16.7 |
| LJMUCV390 | 8 | 4324 | 1.91 | 23.7 |
| LJMUCV391 | 8 | 4328 | 1.94 | 55.2 |
| LJMUCV392 | 7 | 4312 | 1.82 | 21.1 |
| LJMUCV393 | 8 | 4318 | 1.75 | 31.9 |
| LJMUCV394 | 8 | 5194 | 2.07 | 47.4 |
| LJMUCV395 | 8 | 5184 | 1.97 | 77.9 |
| LJMUCV396 | 7 | 4316 | 1.71 | 32.3 |
| LJMUCV397 | 8 | 4326 | 2.08 | 18.7 |
| LJMUCV398 | 8 | 4312 | 1.84 | 23.5 |
| LJMUCV399 | 8 | 4304 | 1.85 | 27.2 |
| LJMUCV400 | 9 | 4326 | 2.03 | 9.77 |
| LJMUCV401 | 9 | 4336 | 2.07 | 46.6 |
| LJMUCV402 | 8 | 4320 | 1.96 | 16.5 |
| LJMUCV403 | 8 | 4324 | 1.95 | 24.9 |
| LJMUCV404 | 8 | 5158 | 2.03 | 19.1 |
| LJMUCV405 | 4 | 5158 | 2.03 | 33.8 |
| LJMUCV406 | 5 | 5168 | 1.91 | 10.1 |
| LJMUCV407 | 8 | 4330 | 1.59 | 38.4 |
| LJMUCV408 | 9 | 4312 | 1.96 | 53.2 |
| LJMUCV409 | 5 | 5144 | 2.09 | 23.6 |
| LJMUCV410 | 9 | 5182 | 1.87 | 15.8 |
| LJMUCV411 | 9 | 4316 | 1.93 | 65.3 |
| LJMUCV412 | 9 | 4326 | 1.94 | 13.2 |
| LJMUCV413 | 8 | 4318 | 2.01 | 163 |
| LJMUCV414 | 9 | 4306 | 1.82 | 14.3 |
| LJMUCV415 | 8 | 4322 | 1.76 | 23.7 |
| LJMUCV416 | 5 | 5204 | 1.88 | 29.2 |
| LJMUCV417 | 6 | 4310 | 2.22 | 37.0 |
| LJMUCV418 | 7 | 4278 | 2.14 | 13.3 |
| LJMUCV419 | 4 | 5168 | 2.42 | 26.6 |
| LJMUCV420 | 9 | 4314 | 1.84 | 9.45 |
| LJMUCV421 | 9 | 4324 | 2.10 | 144 |
| LJMUCV422 | 7 | 4340 | 1.86 | 17.3 |
| LJMUCV423 | 8 | 4328 | 1.73 | 25.9 |
| LJMUCV424 | 9 | 5156 | 1.75 | 45.6 |
| LJMUCV425 | 8 | 4308 | 1.60 | 51.0 |
| LJMUCV426 | 8 | 4312 | 1.32 | 25.1 |
| LJMUCV427 | 8 | 4340 | 1.78 | 32.7 |
| LJMUCV428 | 8 | 5170 | 1.82 | 8.37 |
| LJMUCV429 | 6 | 5112 | 1.71 | 17.3 |
| LJMUCV430 | 7 | 7024 | 1.94 | 19.2 |
| LJMUCV431 | 6 | 5114 | 1.82 | 2.05 |
| LJMUCV432 | 8 | 5184 | 1.92 | 26.5 |
| LJMUCV433 | 7 | 5182 | 1.89 | 35.3 |
| LJMUCV434 | 9 | 5196 | 2.32 | 17.2 |
| LJMUCV435 | 9 | 4312 | 1.95 | 85.1 |
| LJMUCV437 | 9 | 5168 | 1.83 | 17.8 |
| LJMUCV438 | 9 | 4288 | 1.79 | 12.8 |
| LJMUCV439 | 8 | 5168 | 1.98 | 25.3 |
| LJMUCV440 | 7 | 4310 | 1.70 | 11.1 |
| LJMUCV441 | 8 | 4326 | 1.80 | 44.2 |
| LJMUCV442 | 7 | 4296 | 1.78 | 11.4 |
| LJMUCV443 | 8 | 5144 | 1.85 | 20.5 |
| LJMUCV444 | 8 | 5206 | 1.91 | 11.5 |
| LJMUCV445 | 9 | 5210 | 1.88 | 28.2 |
| LJMUCV446 | 9 | 5188 | 2.09 | 46.1 |
| LJMUCV447 | 9 | 5172 | 1.64 | 47.4 |
| LJMUCV448 | 8 | 5190 | 1.90 | 21.7 |
| LJMUCV449 | 9 | 4310 | 2.05 | 71.7 |
| LJMUCV450 | 5 | 5188 | 1.64 | 22.8 |
| LJMUCV451 | 9 | 4318 | 1.96 | 34.1 |
| LJMUCV452 | 8 | 4324 | 1.79 | 57.9 |
| LJMUCV453 | 9 | 4370 | 1.80 | 255 |
| LJMUCV454 | 8 | 4330 | 1.81 | 16.2 |
| LJMUCV455 | 9 | 4416 | 1.93 | 50.1 |
| LJMUCV456 | 9 | 4308 | 2.04 | 40.9 |
| LJMUCV457 | 9 | 4320 | 1.53 | 12.8 |

N: number of peaks; SNR: signal to noise ratio
